# EAG1 channel is a central regulator of tumor growth and metastasis in A549 non-small cell lung cancer cells

**DOI:** 10.1016/j.jbc.2026.113590

**Published:** 2026-09-23

**Authors:** Joos Berghausen, Emre Deniz, Ze-Jun Wang, Marcel Schmidt, Junfeng Ma, Karen Creswell, Aykut Üren, Eric Glasgow, Anton Wellstein, Tinatin I. Brelidze

**Affiliations:** 1 Department of Pharmacology and Physiology, Georgetown University Medical Center, Washington, District of Columbia, USA; 2 Department of Oncology, Georgetown University Medical Center, Washington, District of Columbia, USA

**Keywords:** KCNH1, EAG, NSCLC, A549, EMT, cell cycle, xenograft, zebrafish

## Abstract

The voltage-gated potassium channel Ether-à-go-go 1 (EAG1) is aberrantly expressed in many cancers, yet its mechanistic contributions to tumor growth and metastasis in non–small cell lung cancer (NSCLC) remains poorly defined. Here, we establish a genetic EAG1 knockout in A549 NSCLC cells and demonstrate that loss of EAG1 markedly suppresses tumor growth and metastasis using a combination of tissue culture, xenograft, and multi-omics approaches. EAG1 knockout reduced proliferative ability across multiple *in vitro* assays and *in vivo* zebrafish xenografts. Furthermore, loss of EAG1 induced G1-phase cell-cycle arrest, accompanied by broad suppression of cell cycle regulators. Additionally, knocking out EAG1 significantly reduced migratory and invasive properties *in vitro* and markedly impaired extravasation *in vivo*. Integrated transcriptomic and proteomic analyses revealed that EAG1 regulates E2F signaling and epithelial–mesenchymal transition (EMT). Consistent with this, EAG1 knockout attenuated transforming growth factor-beta–induced EMT by disrupting the cadherin switch, a key mechanism for EMT, and abolished EMT-driven migration, demonstrating that EAG1 is required for the acquisition of a migratory phenotype. Together, these findings establish EAG1 as a central regulator of proliferation and metastasis of A549 cells, highlighting EAG1 as a potential therapeutic target in NSCLC.

Ether-à-go-go 1 (EAG1), also known as KCNH1 and Kv10.1, is a voltage-gated potassium channel encoded by KCNH1 gene with well-established oncogenic properties (1, 2, 3). While in healthy individuals, EAG1 expression is largely confined to the central nervous system, EAG1 is aberrantly overexpressed in a wide range of human cancers (4, 5, 6, 7, 8, 9). Its expression correlates with increased tumor growth and aggressiveness. Functional studies across multiple cancer models have shown that inhibition of EAG1 channel activity with RNAi and EAG1-specific antibody blockers reduces tumor growth both *in vitro* and *in vivo* (2, 10), positioning EAG1 as a potential cancer-specific therapeutic target. Moreover, transfection of noncancerous Chinese hamster ovary and NIH3T3 fibroblast cells with EAG1 induces a malignant phenotype, while knocking down EAG1 expression suppresses cancer-associated properties, such as proliferation (1, 2), indicating that EAG1 is a major driver of tumorigenesis. Importantly, global knockout of EAG1 is well tolerated in mice, with no anatomical or behavioral deficits, further supporting EAG1’s potential as a cancer-selective therapeutic target (11).

Tumor progression is driven by dysregulation of cell-cycle control mechanisms that allow uncontrolled cell division by bypassing growth checkpoints and evading tumor suppressive pathways (12). In parallel, the acquisition of migratory and invasive properties enables cancer cells to disseminate from the primary tumor and colonize distant organs, a multistep process that dramatically worsens treatment outcomes (12). Uncontrolled cell proliferation and metastasis are defining hallmarks of cancer and are responsible for the majority of cancer-related mortality (13). Non–small cell lung cancer (NSCLC) is considered one of the most deadly cancers, with 135,000 deaths annually (14). Therefore, there is an urgent need for identification of novel therapeutic targets for NSCLC treatment. Here, we looked at the role of EAG1 channels in NSCLC and therapeutic potential of inhibiting EAG1 activity for NSCLC treatment.

The cellular pathways through which EAG1 channels promote cancer progression are not fully understood and appear to involve multiple cancer type–specific mechanisms. EAG1 has been shown to enhance proliferation of hepatocellular carcinoma cells by inhibiting the degradation of SKP2, a negative regulator of cell-dependent kinase (CDK) inhibitors and a key regulator of cell cycle progression (15, 16). Another previously reported link between EAG1 and cell cycle is E2F1, a central regulator of cell-cycle progression (17). It has been shown in cervical cancer that an increase in E2F1 expression increases EAG1 expression, indicating that EAG1 is a direct transcriptional target of E2F1 (18, 19). EAG1 has also been implicated in angiogenic signaling, as silencing EAG1 in osteosarcoma cells suppresses proliferation and migration through the VEGF pathway (20, 21, 22). Additionally, EAG1 expression is upregulated *via* ERK1/2 signaling under serum-deprived conditions in A549 NSCLC lung cancer cells, suggesting a role in promoting survival within nutrient-poor tumor microenvironments (23). The EAG1-associated cellular mechanisms appear to be cancer-type dependent. For instance, genetic suppression of EAG1 induces G1-phase cell cycle arrest in gastric cancer cells and G2-phase arrest in cervical cancer cells, suggesting that EAG1-mediated regulation of cell proliferation may vary across cancer types and underscores the importance of characterizing EAG1-dependent cellular pathways within individual tumor types (19, 24).

Pharmacological inhibition or genetic suppression of EAG1 reduces migration and invasion in several cancer types. Knocking down EAG1 in osteosarcoma and hepatocellular carcinoma cells leads to a decrease in migration and invasion (15, 20). Despite the strong evidence of a major role of EAG1 in metastasis, studies of cellular pathways responsible for this role of EAG1 are limited. A few studies report a link between EAG1 and cytoskeletal regulation in noncancerous Chinese hamster ovary cells by demonstrating colocalization of actin and EAG1 and modulation of EAG1 currents by actin filaments (25, 26). EAG1 has been shown to enhance the migration of triple-negative breast cancer cells by influencing Ca^2+^ entry through Orai1 channels (27). It has also been reported that EAG1 expression is upregulated during transforming growth factor-beta (TGF-β)–induced epithelial–mesenchymal transition (EMT) in A549 NSCLC cells, a critical step in metastasis (28). However, despite increased EAG1 expression during EMT, it is not known if EAG1 is a regulator in the EMT pathways or is overexpressed as a consequence of EMT. Additionally, pharmacological inhibition of EAG1 with astemizole failed to block EMT-associated migration, leaving the functional significance of EAG1 in EMT unresolved (28). Together, these observations highlight substantial gaps in our understanding of EAG1’s role in metastasis, particularly its contribution to EMT, and emphasize the need for further mechanistic investigation.

Although prior studies establish EAG1 as an important player in tumorigenesis, elucidation of its exact contribution in long-term tumor progression has been hindered by a lack of appropriate experimental tools, such as EAG1-knockout cancer cell lines and EAG1-selective small-molecule inhibitors. With the exception of a recent study using an EAG1-knockout breast cancer cell line (29), inhibition of EAG1 channel activity had been attempted using RNAi, nonspecific small-molecule inhibitors, and EAG1-specific antibodies (2, 10, 30). Each of these approaches carries notable limitations (5, 31). For instance, RNAi only temporarily reduces protein expression, making it impossible to investigate the long-term effects of sustained EAG1 silencing or to utilize *in vivo* xenograft models. Furthermore, known EAG1 inhibitors, including astemizole and chlorpromazine, exhibit strong off-target inhibition of human ether-à-go-go–related gene (hERG1) channels, which are also known to contribute to tumorigenesis (5, 32, 33, 34). Therefore, acute pharmacological blockade cannot isolate EAG1-specific functional contributions from hERG1-mediated effects. Finally, the restricted accessibility and penetration of antibodies may paint an incomplete picture of EAG1-dependent effects in cancer. Therefore, genetic ablation of EAG1 is a much-needed approach to further gain insight into the role of EAG1 in tumorigenesis.

Here, we investigated the oncogenic role of EAG1 in A549 NSCLC cells by assessing the functional consequences of genetic silencing of EAG1 using the CRISPR technology on tumor growth and metastatic behavior both *in vitro* and *in vivo*. Genetic ablation of EAG1 significantly reduced tumor growth across multiple *in vitro* cell proliferation assays and in zebrafish xenograft models. In parallel, EAG1 knockout markedly impaired cancer cell migration and invasion *in vitro*, findings that were further supported by reduced extravasation *in vivo*, a critical step in metastasis. To elucidate the underlying mechanisms, we performed integrated transcriptomic and proteomic analyses, which revealed that EAG1 is involved in E2F and EMT signaling. Our results indicate that loss of EAG1 in A549 cells induces G1-phase cell-cycle arrest and disrupts EMT-associated signaling. Notably, we demonstrate for the first time that EAG1 plays a major role in EMT-driven migration through the regulation of the N-cadherin/E-cadherin switch, a key EMT marker.

## Results

### Generation and verification of EAG1-knockout in A549 cells

To investigate the oncogenic role of EAG1 channels in NSCLC, EAG1 was knocked out in A549 cells, a widely used NSCLC model (35), using CRISPR/Cas9 technology. gRNAs were designed to target Exon 6 of KCNH1 gene so that Cas9 can excise 61 bps region (gRNA1: CCCTGCTGGTCCAACAAAGGTGG and gRNA2: GCATGAACTACCTGAAGACGTGG) (Fig. 1*A*). EAG1 channels are assembled from four subunits, with each containing six transmembrane segments (S1-S6) (36). Segments S5 and S6 of all four subunits form the centrally located pore of the channel that allows potassium ion flux down their electrochemical gradient (37). Therefore, a frame-shift mutation upstream of the S5-S6 would prevent formation of a functional EAG1 channel. The Cas9-mediated excision is in the region encoding the intracellular loop between S2 and S3 transmembrane segments of EAG1 channels and is expected to cause a frameshift mutation and a premature stop codon upstream of the pore domain, rendering the channel nonfunctional.

The successful EAG1 gene knockout was confirmed *via* Sanger sequencing of cDNA, which was converted from the isolated RNA (Figure S1). Analysis of the sequence chromatograms unequivocally confirmed the precise excision of the 61 nucleotides within exon 6 in the EAG1-KO cell line, validating the frameshift mutation at the base-pair level. Additionally, the success of the knockout was further confirmed by polymerase chain reaction (PCR) using the forward and reverse primers annealing outside the expected deletion region (F1/R1) with the PCR product length of 766 base pairs in the absence of the excision, and the forward primer annealing within the deleted region and the reverse primer annealing outside of the region (F2/R1) with the PCR product length of 373 base pairs in the absence of the excision (Fig. 1*A*). PCR amplification using primers annealing outside of the deletion site revealed a clear reduction in the amplified product length between WT and EAG1-KO cells, consistent with the excision of 61 bp (Fig. 1*B*). In contrast, amplification using a forward primer annealing within the deleted region and a reverse primer annealing outside of the deleted region produced a detectable PCR product only in WT cells, confirming successful gene deletion at the transcript level (Fig. 1*B*). Consistent with these findings, Western blot analysis demonstrated a marked reduction in EAG1 protein expression in EAG1-KO cells compared to WT control, confirming efficient knockout at the protein level. A representative Western blot image with bands corresponding to EAG1 and actin, and the corresponding quantitative analysis are shown in Figure 1*C*, while the images of uncropped Western blots for all three repeated experiments are shown in Figure S2. Additionally, reverse transcription-quantitative PCR (RT-qPCR) experiments revealed that EAG1 mRNA levels were substantially reduced in EAG1-KO cells compared to the WT control, indicating that the genetic modification diminishes both EAG1 protein expression and transcript levels (Fig. 1*D*).

**Figure 1:**
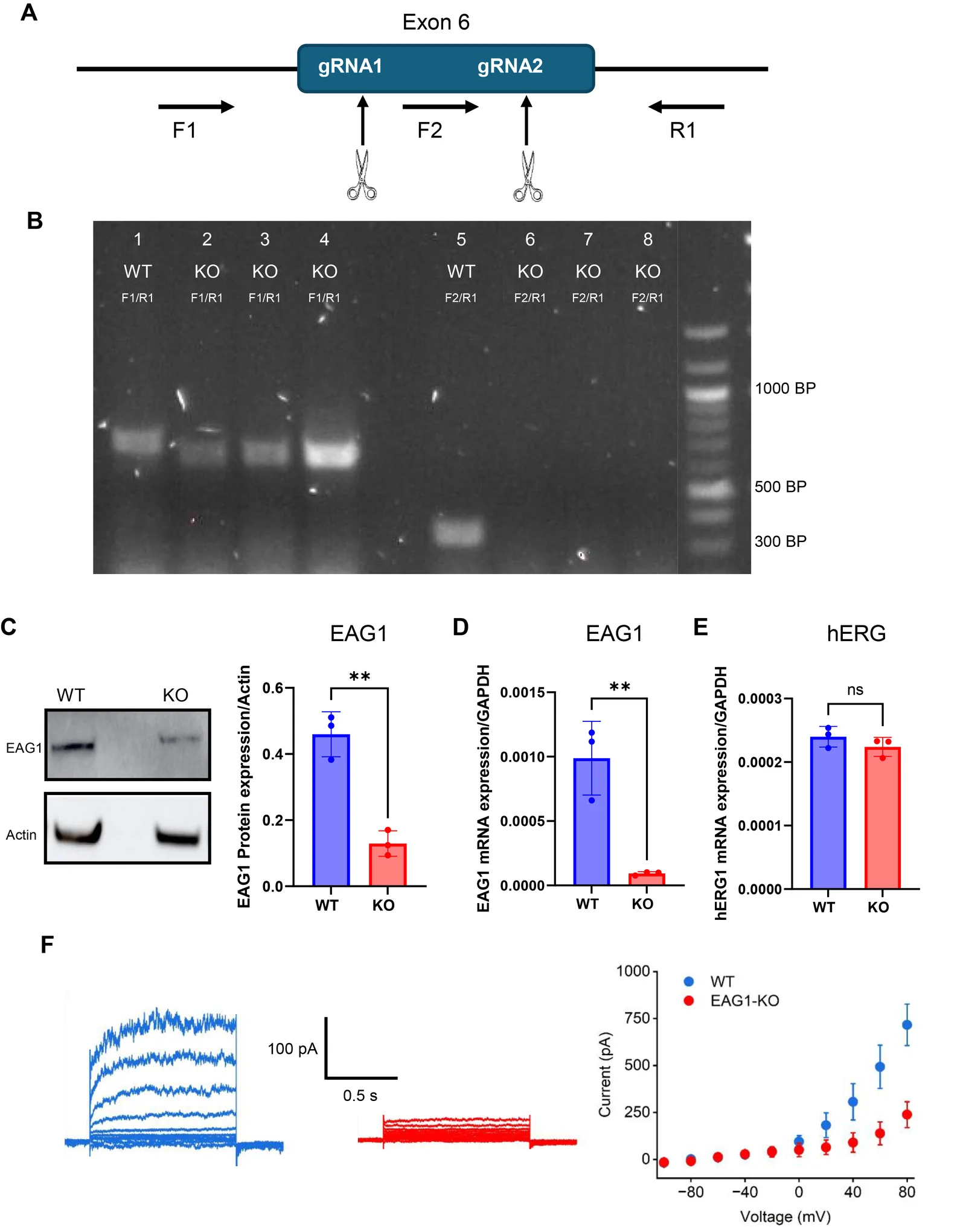
**Generation and verification of EAG1 KO A549 cells.***A*, schematics of gRNAs targeting exon 6 of KCNH1 gene so that Cas9 can excise 61 bps region, causing a frameshift mutation. *B*, agarose gel of PCR reaction for primers F1 and R1 (lanes 1–4) annealing outside of the excised region for WT and EAG1-KO A549 cell lysates, as indicated, and F2 and R1 primers (lanes 5–8) with primers F2 annealing within the excised region and R1 annealing outside the excised region. The PCR reactions were repeated three times for EAG1-KO cells for each primer set, as shown. *C,* representative Western blot images of the WT and EAG1-KO A549 cell lysates stained with EAG1 channel-specific antibody and quantitative analysis for three repeats. Actin staining was used as a positive control. *D, E,* bar graphs of EAG1 (*D*) and hERG1 (*E*) mRNA expression for WT and EAG1-KO A549 cells relative to GAPDH expression assessed with RT-qPCR. The experiments were repeated three times for each cell line. *F*, representative whole-cell currents recorded from WT (*blue)* and EAG1-KO (*red*) A549 cells and steady-state current *versus* voltage plots for WT and EAG1-KO A549 cells. The steady-state currents were measured at the end of the corresponding voltage pulse and averaged for n = 4 for WT and n = 4 for EAG1-KO different A549 cells. The data are presented as mean ± SD, and unpaired Student’s *t* test is used to determine statistical significance throughout the figure (∗∗*p* < 0.01; ns *p* > 0.05). EAG1, Ether-à-go-go 1; GAPDH, glyceraldehyde-3-phosphate dehydrogenase; hERG1, human ether-à-go-go–related gene.

hERG1 channels, encoded by *KCNH2* gene, are members of the same potassium-selective channel family as EAG1 and have been also implicated in cancer (38, 39, 40, 41). To validate that the expression of hERG1 does not compensate for the loss of EAG1, we performed qPCR experiments to determine the expression of hERG1 in both WT and EAG1-KO cells. There was no significant difference in hERG1 mRNA expression relative to glyceraldehyde-3-phosphate dehydrogenase (GAPDH) between WT and EAG1-KO A549 cells, demonstrating no compensatory change in hERG1 levels as a result of EAG1 knockout (Fig. 1*E*).

To investigate the effect of EAG1 channel knockout on endogenous potassium currents in A549 cells, we recorded whole-cell currents in WT and EAG1-KO A549 cells. WT A549 cells exhibited robust depolarization-activated potassium currents, while these currents appear to be substantially smaller in EAG1-KO A549 cells (Fig. 1*F*). These electrophysiological results demonstrate that EAG1 forms a functional channel in A549 cells, and the EAG1 knockout diminishes EAG1-mediated currents.

In addition to EAG1 and hERG1 potassium channels, several other potassium channels, including Kv1.3 channels encoded by *KCNA3* gene, Kir3.1 (GIRK1) encoded by *KCNJ3*, K2P9.1 (TASK-3) encoded by *KCNK9*, KCa3.1 (IK1, SK4) encoded by *KCNN4*, and BK (Slo1) encoded by *KCNMA1*, have been implicated in oncogenesis (42, 43, 44, 45, 46). To evaluate whether EAG1 knockout alters the expression levels of these targets, we conducted RT-qPCR (Figure S3). Our results revealed that EAG1 ablation significantly increases *KCNA3* transcript levels, while significantly decreasing *KCNMA1* and *KCNN4* transcripts. The changes in the *KCNJ3* and *KCNK9* transcript levels between WT and EAG1-KO cells were not statistically significant. Since the channels encoded by *KCNA3* are typically pro-oncogenic, an increase in their abundance cannot account for the diminished tumorigenic drive observed following EAG1 knockout. The downregulation of *KCNMA1* and *KCNN4* demonstrates that there is no compensatory overexpression of the corresponding channels in response to EAG1 deletion. We cannot rule out the possibility that a portion of the antiproliferative or antimetastatic phenotypes observed in our study may stem from the decreased expression of *KCNMA1* or *KCNN4* resulting from EAG1 ablation. Overall, the downregulation of *KCNMA1* and *KCNN4* channels suggests that EAG1 knockout alters broader transcriptional networks, positioning EAG1 as an upstream regulator that orchestrates a broader oncogenic channel signaling network in A549 lung cancer cells. Crucially, previous studies that established the oncogenic roles of KCNMA1 and KCNN4 did not evaluate EAG1 expression. It is entirely plausible that the protumorigenic effects previously attributed to KCNMA1 or KCNN4 are functionally coupled to, or downstream of, EAG1 signaling. Taken together, our study highlights an interconnected channel network, providing a compelling foundation for future follow-up investigations.

### EAG1 channels enhance A549 cell growth in vitro

EAG1 channels have been widely reported to exhibit oncogenic properties, with knockdown studies demonstrating reduced tumor growth both *in vitro* and *in vivo* across multiple cancer models (1, 2, 15, 20, 21). However, to our knowledge, the role of EAG1 has not previously been examined using genetic knockdown or knockout approaches in A549 cells. To investigate the contribution of EAG1 to A549 cell growth *in vitro*, we seeded WT and EAG1-KO A549 cells into 6-well plates and counted the cells 24 and 72 h after seeding. The 48-h window was chosen for manual counting as at this time point the growth rate divergence was clear; however, complete confluence had not yet been reached. Cell counting revealed a significantly greater increase in the number of WT cells compared to EAG1-KO cells, with the 3.58 ± 0.35-fold increase in WT cell number and only 2.52 ± 0.63-fold increase in EAG1-KO cells over the 48 h period (Fig. 2*A*). To further evaluate the role of EAG1 channels in A549 cell growth, we used the CellTiter-Blue cell viability assay. WT and EAG1-KO A549 cells were seeded into 96-well plates, and their viability was measured 0, 24, and 48 h after seeding (Fig. 2*B*). No significant difference in cell viability between WT and EAG1-KO cells was observed 0 h after seeding, confirming equal initial seeding density. By 24 h, however, the viability of EAG1-KO cells was 0.841 ± 0.046-fold reduced compared to WT cells. This reduction persisted at 48 h, with the viability of EAG1-KO cells 0.814 ± 0.04-fold reduced compared to WT cells.

**Figure 2:**
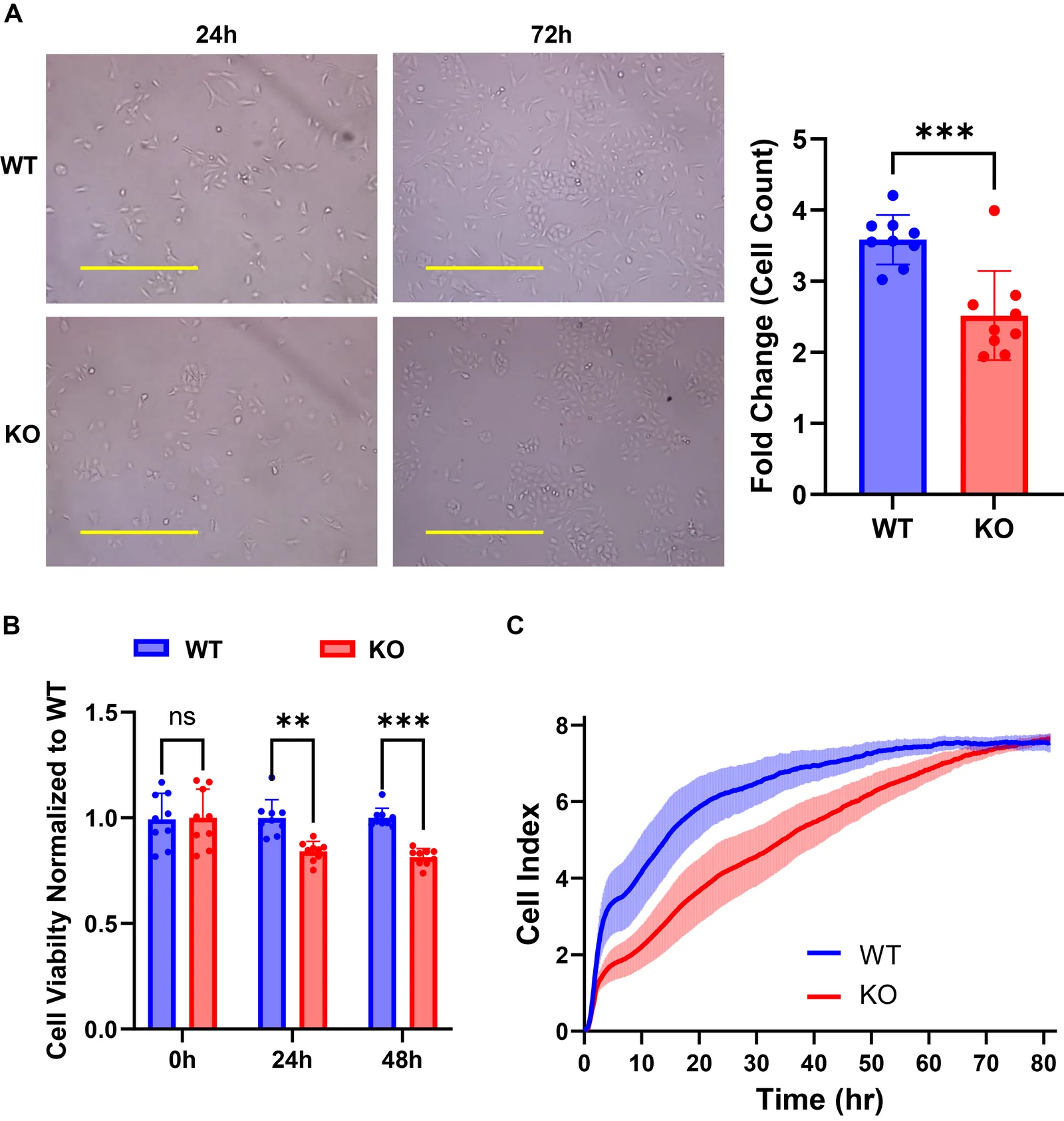
**Knocking out EAG1 decreases cell growth *in vitro*.***A, left*, representative images of A549/WT cells top and A549/EAG1-KO cells bottom, imaged at 24 and 72 h after plating. Scale bar = 0.5 mm. *A, right*, bar graph of the fold change in the cell count from 24 h to 72 h after plating. Data represent three repeated experiments, with three representative images acquired for each cell line. *B*, viability of A549/EAG1-KO cells (*red*) relative to the viability of A549/WT cells (*blue*) 0 h, 24 h, and 48 h after the cell plating. Data represent three repeated experiments for each cell line. *C*, real time cell growth curve monitored with xCELLigence system for A549/WT (*blue*) and A549/EAG1-KO (*red*) cells from 0h to 80 h. Data represent four repeated experiments, with each having two technical replicates for each cell line. The data are presented as mean ± SD throughout the figure. Unpaired Student’s *t* test was used for (*A*), and one-way ANOVA was used for (*B*) to determine statistical significance (∗∗∗*p* < 0.001; ∗∗*p* < 0.01; ns *p* > 0.05). EAG1, Ether-à-go-go 1.

To characterize the effect of EAG1 on A549 cell growth dynamics in real time, we continuously monitored WT and EAG1-KO A549 cell proliferation using the xCELLigence system, which uses impedance measurements for the real-time monitoring of cell growth and migration. WT and EAG1-KO cells were seeded into the 16-well electronic microtiter plate (E-Plate). The E-Plate has gold microelectrodes in the base that are connected to a computer, allowing the measurements of the impedance with the electrical circuit. The continuous impedance measurements are converted into cell index (CI), a quantitative measure of cell number in each well. These experiments revealed a pronounced and sustained differences in the cell growth rate between WT and EAG1-KO cells monitored in real-time between 0 and 80 h after seeding (Fig. 2*C*). WT cells reached confluence at approximately 40 h, whereas EAG1-KO cells did not reach confluence until roughly 70 h, at which point growth rates converged for the two cell lines. Taken together, these complementary assays demonstrate that EAG1 channels play a critical role in promoting A549 cell growth, with genetic ablation of EAG1 leading to a significant reduction in tumor growth *in vitro*.

### EAG1 channel knockout decreases tumor growth of A549 cells in vivo

To evaluate the role of EAG1 channels in A549 cell *in vivo,* we used xenografts in zebrafish. DiI-labeled WT or EAG1-KO A549 cells were implanted into the yolk sac of zebrafish larvae at 2 days postfertilization (dpf) (Fig. 3*A*), and tumor size was quantified immediately after implantation (Day 0) and 72 h later (Day 3) (Fig. 3*B*). Over the 3-day period, tumors derived from WT cells exhibited a substantial increase in size, expanding by 95.88 ± 65.56% on average, whereas tumors formed by EAG1-KO cells increased by only 59.34 ± 54.46% on average (Fig. 3*C*). This reduction in tumor growth was statistically significant and corroborates our results for the effect of EAG1 knockout on the cell growth *in vitro*. Collectively, our *in vitro* and *in vivo* findings demonstrate that loss of EAG1 markedly impairs A549 tumor growth, underscoring a critical role for EAG1 channels in driving the proliferative ability of A549 cells.

**Figure 3:**
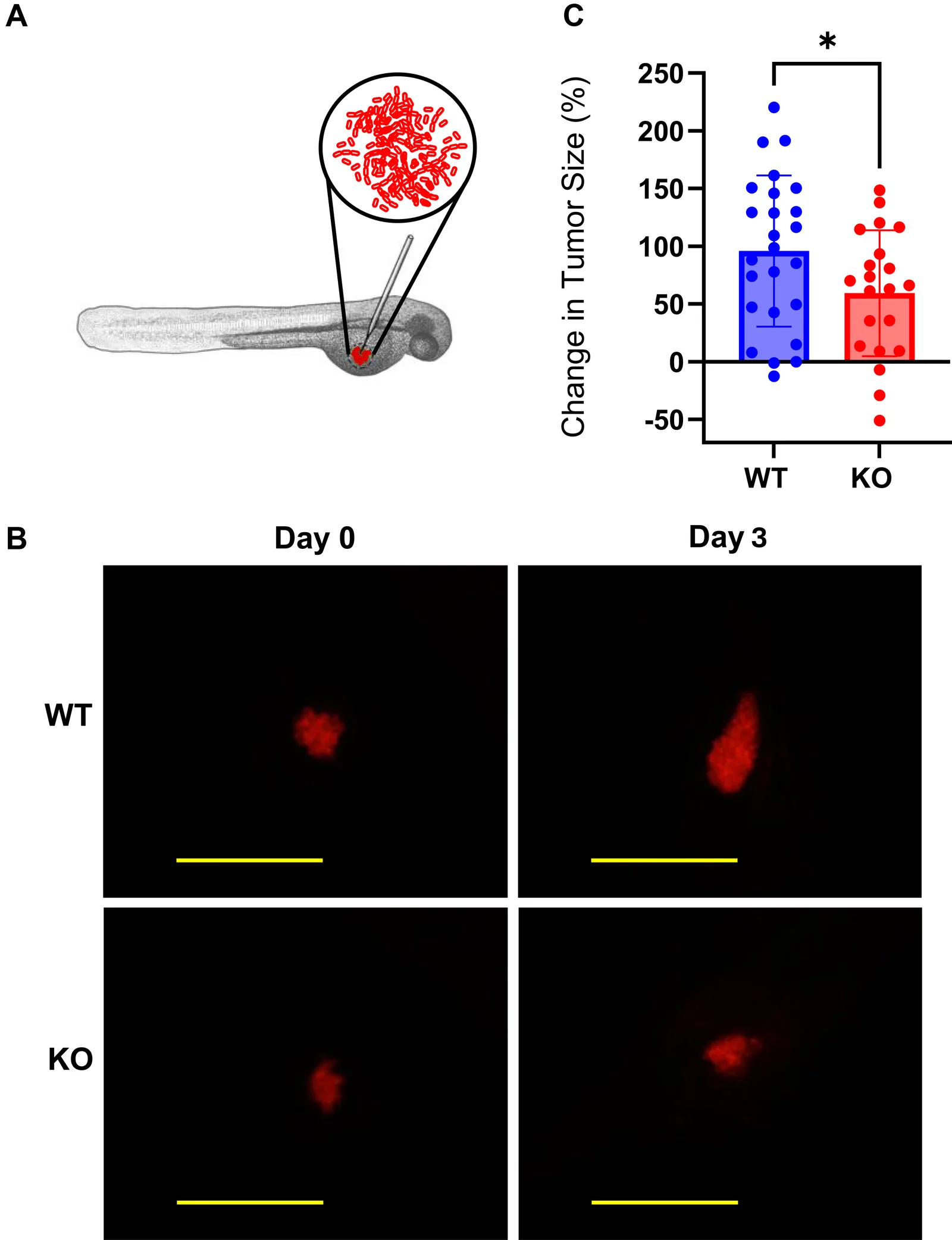
**Knocking out EAG1 decreases cell growth *in vivo*.***A*, schematic of the experimental design of injecting DiI-labeled cells into the yolk-sac of 2dpf zebrafish. *B*, representative image of a zebrafish xenograft with DiI-labeled A549/WT cells (*top*) and A549/EAG1-KO cells (*bottom*) on Day 0 and Day 3 after the cell injection. Scale = 0.5 mm. *C*, quantification of % changes in tumor area on Day 3 after the implantation of A549/WT cells (*blue*) and A549/EAG1-KO cells (*red*). Number of zebrafish embryos N = 25 for WT A549 cell xenografts, and N = 21 for EAG1-KO A549 cell xenografts. The data are presented as mean ± SD. Unpaired Student’s *t* test analysis was used to determine statistical significance (∗*p* < 0.05). EAG1, Ether-à-go-go 1.

### EAG1 channel knockout leads to G1-phase cell cycle arrest

Previous studies have implicated EAG1 channels in cell cycle regulation, although their specific effects appear to be tumor dependent. For example, EAG1 knockdown induces G2/M-phase arrest in cervical cancer cells, whereas in gastric cancer cells, it results in G1 arrest, suggesting that EAG1-mediated cell cycle control varies by cell type (19, 24). To define the role of EAG1 in cell cycle progression in A549 cells, we performed flow cytometry–based cell cycle analysis in WT and EAG1-KO cells. WT and EAG1-KO A549 cells were seeded and analyzed by flow cytometry 30 h after plating to quantify the proportion of cells in each phase of the cell cycle (Fig. 4*A*). For WT cells, 42.9 ± 0.63% of cells were in G1 phase, 35.04 ± 0.30% in S phase, and 22.07 ± 0.60% in G2/M phase (Fig. 4*B*). In contrast, EAG1-KO cells displayed a distinct redistribution across the cell cycle, with 53.33 ± 0.82% of cells in G1 phase, 30.64 ± 0.25% in S phase, and 16.02 ± 0.57% in G2/M phase (Fig. 4*B*). Quantitative comparison revealed a significant accumulation of EAG1-KO cells in G1 phase, accompanied by corresponding reductions in S and G2/M phases (Fig. 4*B*). These findings indicate that loss of EAG1 induces G1-phase arrest in A549 cells, suggesting that EAG1 channels promote efficient progression through G1.

**Figure 4:**
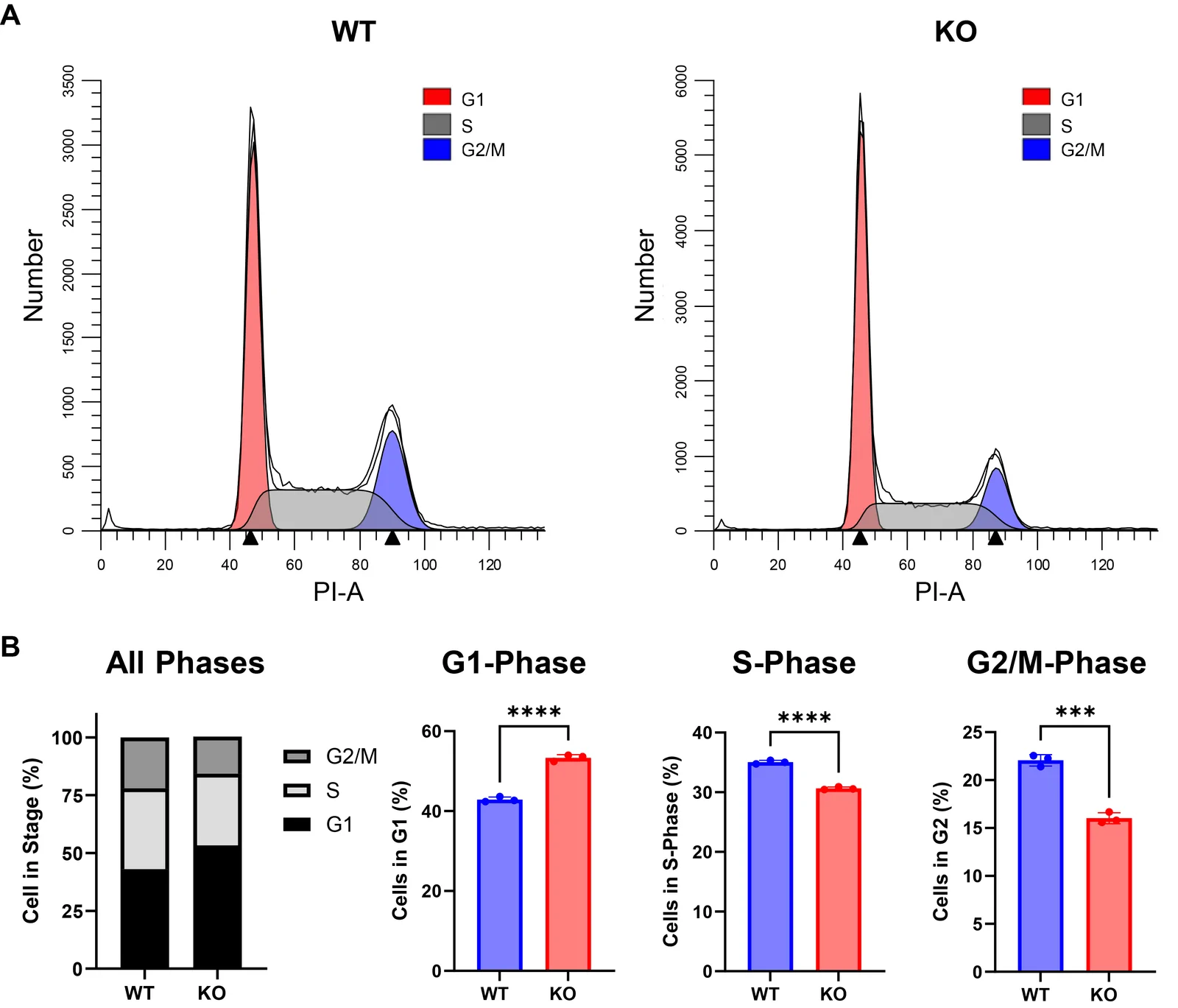
**Knocking out EAG1 leads to G1-phase arrest.***A*, histograms of cell cycle phase distribution of A549/WT cells (*left*) and A549/EAG1-KO cells (*right*) after 30h in culture with G1-phase shown in *red*, S-phase in *gray*, and G2/M-phase in *blue*. *B*, *left*, stacked bar graphs of the % of cells in G1 (*black*), S-phase (*light gray*), and G2/M-phase (*medium gray*) for A549/WT cells and A549/EAG1-KO cells. *B*, *right*, bar graphs comparing the % cells in G1, S, and G2/M cell cycle phases for A549/WT cells (*blue*) and A549/EAG1-KO cells (*red*). Data represent three repeated experiments for each cell. The data are presented as mean ± SD. Unpaired Student’s *t* test analysis was used to determine statistical significance (∗∗∗∗*p* < 0.0001; ∗∗∗*p* < 0.001). EAG1, Ether-à-go-go 1.

### EAG1 channel knockout decreases migration and invasion in vitro

Inhibition of EAG1 channels has been shown to decrease cell migration and invasion in hepatocellular carcinoma, osteosarcoma, and breast cancer cells (15, 20, 27). To assess the contribution of EAG1 to metastatic behavior in NSCLC, we examined differences in migration and invasion between WT and EAG1-KO A549 cells. Cell migration was evaluated using a wound-healing assay, in which wound closure was monitored at 0 and 48 h following wound induction (Fig. 5*A*). After 48 h, WT cells exhibited significantly greater wound closure than EAG1-KO cells, with WT cells achieving 35.02 ± 11.27% wound healing compared to 16.39 ± 5.337% in EAG1-KO cells. These results indicate that EAG1 loss substantially impairs the migratory ability of A549 cells.

**Figure 5:**
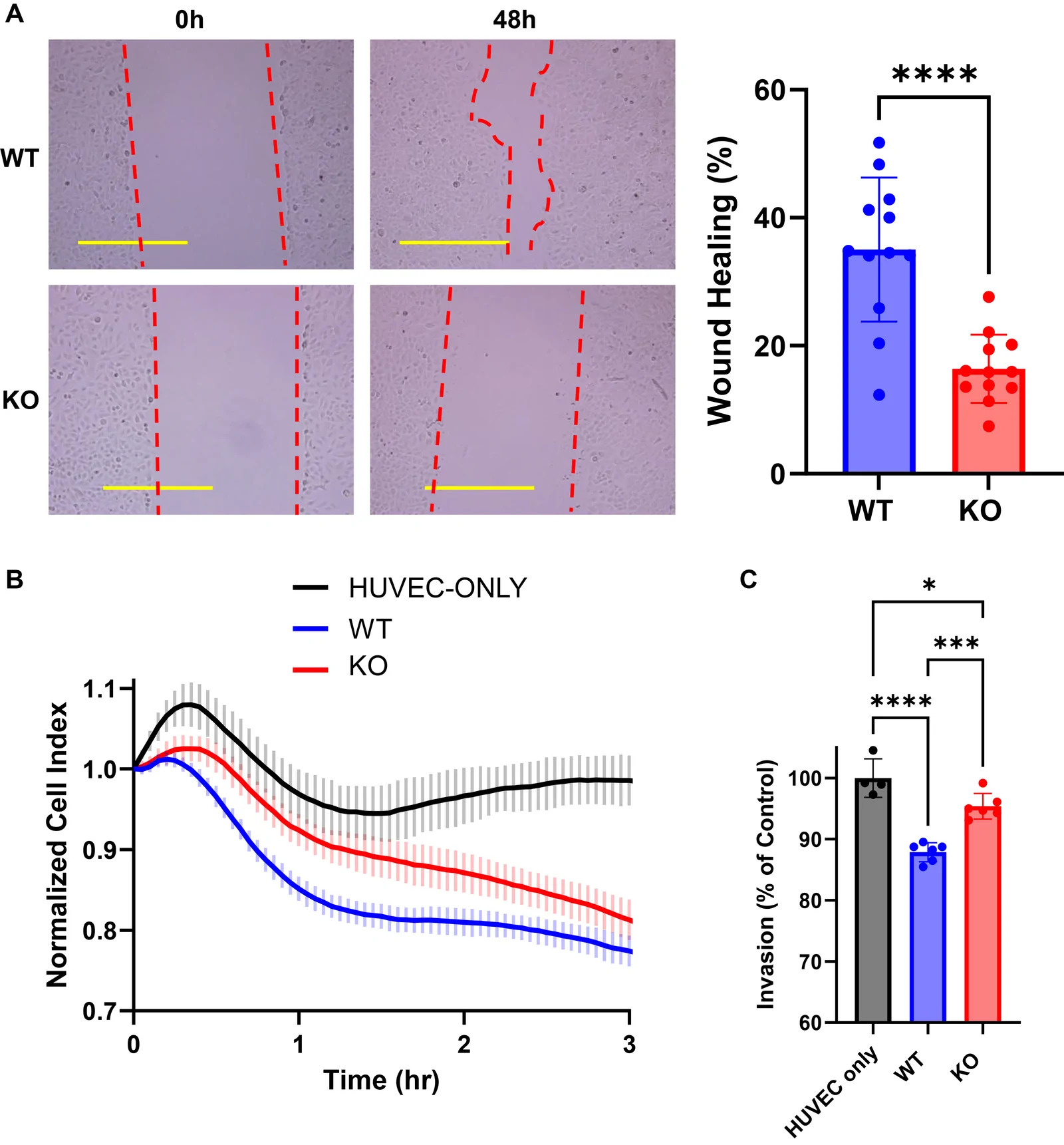
**Knocking out EAG1 decreases migration and invasion *in vitro*.***A*, *left*, representative images of the wound healing of A549/WT cells (*top*) and A549/EAG1-KO cells (*bottom*) at 0 and 48 h after scratch. Scale bar = 0.5 mm. *A*, *right*, bar graph summary of the % wound healing of A549/WT cells (*blue*) and A549/EAG1-KO cells (*red*) at 48 h postscratch. Data represent four repeated experiments, with each having three technical replicates for each cell line. *B*, time-lapse of invading A549/WT cells (*blue*) and A549/EAG1-KO cells (*red*) as measured by the ratio of HUVEC (*black*) layer displacement from 0h to 3h. *C*, bar graph at 1h post plating of the ratio of displaced HUVEC cells (*black*) by A549/WT cells (*blue*) and A549/EAG1-KO cells (*red*). Data represent three repeated experiments, with each having two technical replicates for each cell line. *A, C,* unpaired Student’s *t* test (*A*, *right*) and one-way ANOVA (*C*) was used to determine statistical significance (∗∗∗∗*p* < 0.0001; ∗∗∗*p* < 0.001; ∗*p* < 0.05). The data are presented as mean ± SD throughout the figure. EAG1, Ether-à-go-go 1; HUVEC, human umbilical vein endothelial cell.

To further investigate the role of EAG1 in invasion, we employed the xCELLigence system to monitor A549 cell invasion through an endothelial barrier in real time. xCELLigence system quantifies invasion by measuring the displacement of a human umbilical vein endothelial cell (HUVEC) monolayer by invading tumor cells. EAG1 knockout resulted in a significant reduction in invasive ability compared to WT A549 cells, as evidenced by consistently decreased HUVEC displacement (Fig. 5*B*). For instance, after 1 h, WT cells displaced HUVECs to 87.87 ± 1.56%, whereas EAG1-KO cells displaced HUVECs to only 95.39 ± 2.10% (Fig. 5*C*). Although both cell lines produced statistically significant displacement, EAG1-KO cells exhibited a statistically significantly reduced invasion compared to WT cells (Fig. 5*C*). Collectively, our migration and invasion results demonstrate that EAG1 plays an important role in regulating metastatic traits in A549 cells.

### EAG1 channel knockout decreases extravasation

Metastasis of tumor cells is a multistep process. At first, cancer cells infiltrate into adjacent tissue, followed by cell migration through endothelial cells, into the circulatory system (intravasation). The next steps are the survival of the cancer cells in the circulatory system, followed by migration out of the circulatory system (extravasation), leading to the cancer cell proliferation in a secondary location (47). To assess the role of EAG1 extravasation and intravasation we employed zebrafish xenografts. To examine extravasation, DiI-labeled WT or EAG1-KO A549 cells were injected directly into the circulation of zebrafish larvae *via* the duct of Cuvier at 2 dpf (Fig. 6*A*). Two days postinjection, the number of cells that exited the vasculature and migrated into the tail region was quantified (Fig. 6*B*). This analysis revealed a significant reduction in extravasation events in the absence of EAG1, with 54 of 92 WT-engrafted fish exhibiting extravasation compared to only 27 of 89 fish in the EAG1-KO group (Fig. 6*C*).

**Figure 6:**
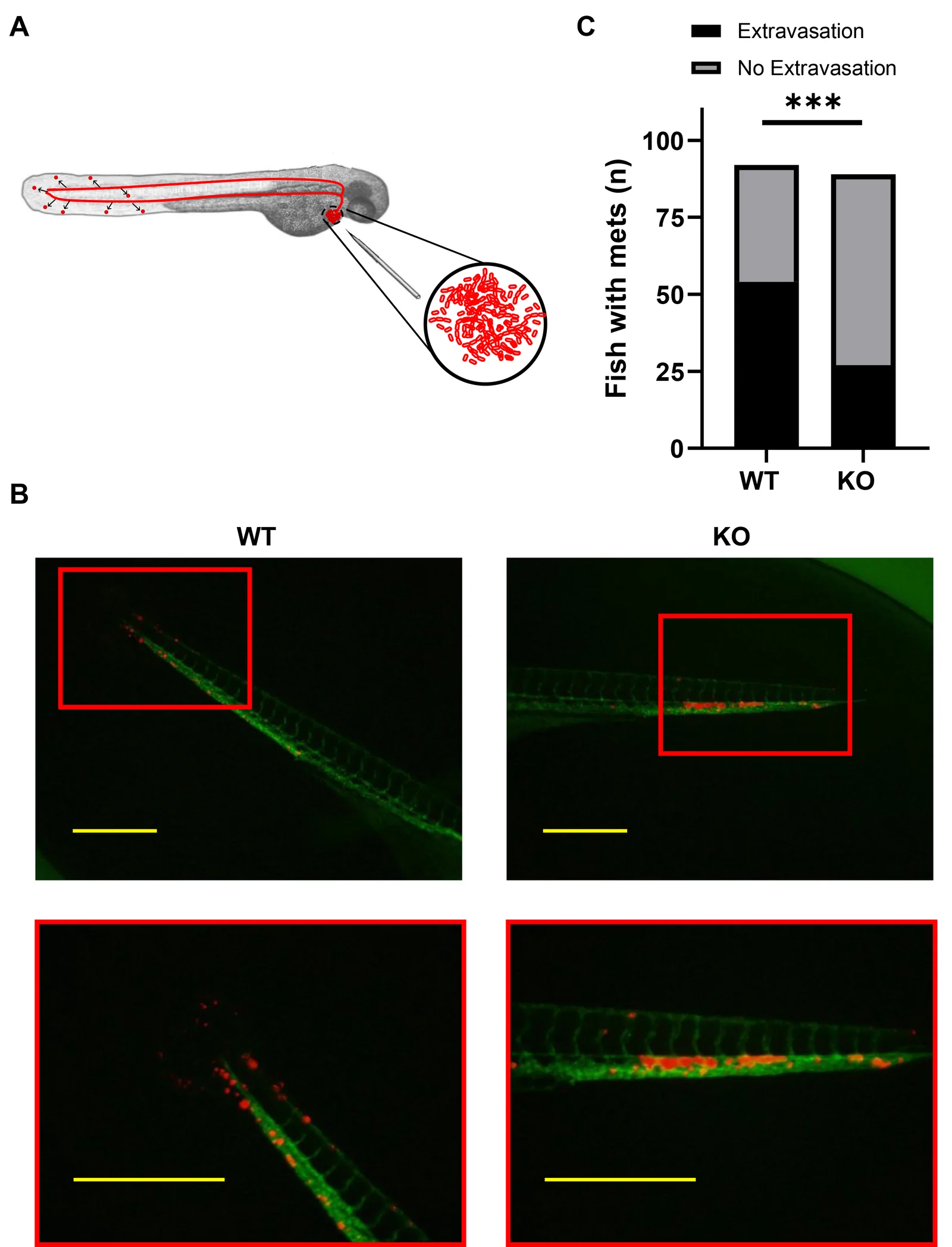
**Knocking out EAG1 decreased the ability for A549 cells to extravasate *in vivo*.***A*, schematic of the experimental design, where DiI-labeled cells are injected into the Duct of Cuvier of zebrafish with GFP-expressing endothelial cells. The cells then travel to the tail region and extravasate out of the circulation and into a secondary tissue. *B*, representative images at 48h postinjection of the tail region of A549/WT group (*left*) and A549/EAG1-KO group at two magnifications. Scale = 0.5 mm. *C*, stacked bar graph showing the amount of fish with at least one extravasating cell (*black*) and no extravasating cells (*gray*) for A549/WT and A549/EAG1-KO xenografts. Number of zebrafish embryos N = 92 for A549/WT xenografts, and N = 89 for A549/EAG1-KO xenografts. A CHI-test was used to determine significance (∗∗∗*p* < 0.001).

We next evaluated intravasation using zebrafish xenografts with WT or EAG1-KO cells implanted into the pericardial cavity of zebrafish embryos (Figure S4*A*). After 2 days, cells that entered the circulation and localized in the vasculature of the tail region were quantified. Xenografts were classified into three discrete bins based on the number of cells that successfully intravasated: 1 to 9 cells, 10 to 29 cells, and >30 cells. The higher the percentage of xenografts in the >30 cells bin, the more aggressive intravasation is. Conversely, the higher percentage of embryos in the lower bin (1–9 cells), the more blunted the intravasation is. No statistically significant difference was observed between unbinned WT and EAG1-KO xenograft groups, although a clear trend toward reduced intravasation was evident for EAG1 knockout (Figure S4*B*; *p* = 0.0927). However, intravasation events in WT-engrafted fish that experienced intravasation were more aggressive, with 8.69% of fish displaying more than 30 intravasating cells, whereas none of the fish engrafted with EAG1-KO A549 cells reached this level of aggressive dissemination (Figure S4*C*). Similarly, 17.39% of WT-engrafted fish exhibited 10 to 29 intravasating cells compared to only 13.33% in the EAG1-KO group. In contrast, low-level intravasation (1–9 cells) was more prevalent in the EAG1-KO group (86.66%) than in WT-engrafted fish (78.26%) (Fig. S4*C*). Taken together, the decreased number of xenografts in the high-cell-number bin reflects a reduced intravasation aggressiveness of EAG1-KO cells *in vivo*.

### EAG1 knockout downregulates cellular pathways associated with cell proliferation and migration

Our results indicate that EAG1 contributes to A549 cell growth and metastasis. To further elucidate the global impact of EAG1 on cellular pathways, we analyzed differential transcript and protein expression for WT and EAG1-KO A549 cells using transcriptomics and proteomics approaches. RNAseq analysis detected a total of 13,035 transcripts. Knocking out EAG1 led to significant upregulation of 293 transcripts and significant downregulation of 361 transcripts (fold change (fc) = log2(−2 to +2), (Fig. 7*A* and Table S1). Our proteomics analysis identified and quantified a total of 6869 proteins. Knocking out EAG1 significantly upregulated 163 proteins and downregulated 209 proteins (fc = log2(−1 to +1)) (Fig. 7*B* and Table S2). For the transcriptomics data, we performed a gene set enrichment analysis (GSEA) to identify pathways that were upregulated or downregulated as a result of knocking out EAG1 (Fig. 7*C*). Our analysis revealed a modest positive enrichment, with the top five upregulated pathways being interferon-alpha-response [normalized enrichment score (NES) = 1.96, false discovery rate (FDR) = 0.003], TNA-alpha signaling *via* NFKB (NES = 1.91, FDR = 0.002), IL6-JAK-STAT3 signaling (NES = 1.9, FDR = 0.001), inflammatory response (NES = 1.88, FDR = 0.001), and complement pathway, which is a component of the innate immune system (NES = 1.68, FDR = 0.008) (Fig. 7*C*). A much more pronounced enrichment was for downregulated pathways. These pathways included cell proliferation pathways in EAG1-KO cells relative to WT cells, including E2F targets (NES = −2.73, FDR = 0.00) (Fig. 7, *C* and *D*), G2M checkpoint (NES = −2.53, FDR = 0.00) (Fig. 7*C*), Myc targets version 1 (NES = −2.47, FDR = 0.00) (Fig. 7*C*), and Myc target version 2 (NES = −2.29, FDR = 0.00) (Fig. 7*C*). Additionally, GSEA identified EMT as one of the most comprehensively disrupted global pathways upon EAG1 knockout (NES = −2.07, FDR = 0.00; Fig. 7, *C* and *E*).

**Figure 7:**
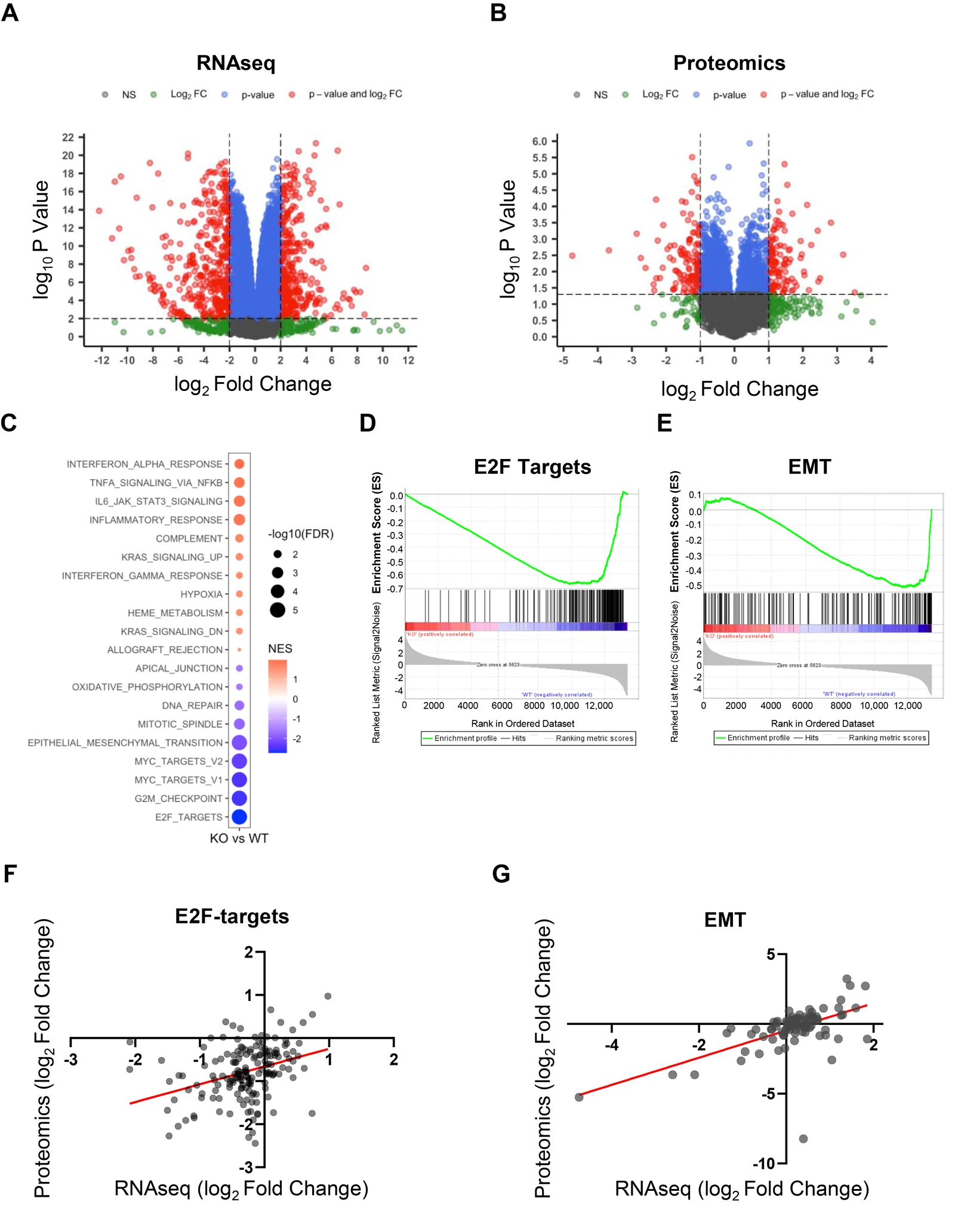
**EAG1-associated pathways revealed with transcriptomic and proteomic analysis.***A, B,* volcano plots of differentially expressed genes (*A*) and proteins (*B*) between A549/EAG1-KO and A549/WT cells, with *vertical dashed lines* representing the absolute log2(FC) threshold of 2 for (*A*) and 1 for (*B*), and *horizontal dashed lines* representing the log10(*p* Value) statistical significance threshold of 2 for (*A*) and 1.3 for (*B*). *Red points* denote data passing both thresholds, *blue points* passing only the statistical significance threshold, *green points* passing FC threshold, and finally *black points* did not pass either threshold. Data represent three repeated experiments for both transcriptomics and proteomics experiments. *C*, bubble plot depicting gene set enrichment analysis (GSEA) for differentially regulated pathways between A549/EAG1-KO and A549/WT cells. Bubble size is proportional to the −log10(FDR), with the scale bar indicating values of 2, 3, 4, and 5. *Bubble color* represents the normalized enrichment score (NES), ranging from negative enrichment (*blue*) to positive enrichment (*red*). *p* values were calculated using the GSEA algorithm relative to a null distribution generated from 1000 permutations. *D, E,* GSEA enrichment plots show negative enrichment for a set of genes downregulating E2F targets (*D*) and EMT (*E*). *F ,G,* correlation plots between the log2(FC) of RNA sequencing and proteomics data for genes involved in the expression of E2F targets (*F*) and genes involved in EMT (*G*). EAG1, Ether-à-go-go 1; EMT, epithelial–mesenchymal transition; FC, fold change.

Importantly, the robust, system-wide downregulation was observed across both the transcriptome and proteome. Correlation analysis indicated that for both E2F and EMT genes, the proteomics data were significantly correlated with the transcriptomics data, with E2F genes having r = 0.35 (95% CI = 0.2022–0.4746) (Fig. 7*F*, Table S3), and EMT having r = 0.59 (95% CI = 0.43–0.71) (Fig. 7*G*, Table S3).

The multi-omics analysis indicated that EAG1 ablation induced a highly coordinated suppression of key components of the cell-cycle machinery (Table 1). Notably, critical drivers of the G1/S transition exhibited robust, concurrent decreases in transcript abundance and protein expression, in agreement with our flow cytometry results (Fig. 4). Analysis of G1 cell-cycle regulators following EAG1 knockout revealed strong transcriptional repression of transcripts for multiple CDKs and cyclins, with partial agreement at the protein level (Table 1). RNA-seq data showed significant downregulation of CDK2 (FC = 0.42, *p* = 3.86 × 10^−8^), CDK4 (FC = 0.69, *p* = 2.06 × 10^−7^), and CDK6 (FC = 0.70, *p* = 1.38 × 10^−6^). Proteomics aligned with this trend for CDK2 (FC = 0.634, *p* = 0.0901) and CDK4 (FC = 0.33, *p* = 0.00237), while for CDK6, protein levels were unchanged (FC = 1.03, *p* = 0.8037). RNA-seq analysis revealed strong suppression of late G1 cyclin E1 (FC = 0.37, *p* = 1.12 × 10^−7^) and cyclin E2 (FC = 0.18, *p* = 9.78 × 10^−7^). Proteomic analysis did not show a significant fold change for cyclin E1 with EAG1 knockout (FC = 0.77, *p* = 0.57) and did not detect cyclin E2 protein levels. Cyclin D1 was also significantly decreased at the RNA level (FC = 0.75, *p* = 2.61 × 10^−7^), with a downward trend in protein expression as well (FC = 0.5, *p* = 0.0677). In contrast, cyclin D3 exhibited no significant transcriptional change (FC = 1.03, *p* = 0.30) but showed a marked and significant reduction at the protein level (FC = 0.29, *p* = 0.00446). It has been well established that correlation between mRNA and protein levels is typically modest due to translational regulation or differences in the half-life of the protein *versus* mRNA (48). This may explain the small discrepancies observed between CDK and cyclin expression at the transcript and protein levels. Nevertheless, both RNAseq and proteomic analyses consistently show that loss of EAG1 reduces expression of key G1 cell-cycle regulators, indicating that EAG1 promotes rapid G1 progression by increasing CDK and cyclin expression.

**Table 1:** Expression of genes and proteins involved in G1/S transition

Gene/Protein	RNA (FC)	RNA (*p*-value)	Protein (FC)	Protein (*p*-value)
CDK2	0.42	3.86 × 10^−8^	0.634	0.0901
CDK4	0.69	2.06 × 10^−7^	0.33	0.00237
CDK6	0.7	1.38 × 10^−6^	1.03	0.8037
Cyclin E1	0.37	1.12 × 10^−7^	0.77	0.57
Cyclin E2	0.18	9.78 × 10^−7^	NA	NA
Cyclin D1	0.75	2.61 × 10^−7^	0.5	0.0677
Cyclin D3	1.03	0.3	0.29	0.00446
P53	1.25	0.0003	NA	NA
MDM2	1.13	0.019	NA	NA
MDM4	1.31	0.00075	NA	NA

The first column lists key analyzed genes and proteins regulating the G1/S transition, including CDK2, CDK4, CDK6, Cyclin E1, Cyclin E2, Cyclin D1, Cyclin D3, P53, MDM2, and MDM4. The second and third columns report the RNA-level fold change (RNA FC) and corresponding statistical significance (RNA *p*-value), respectively. The fourth and fifth columns present the protein-level fold change (Protein FC) and associated *p*-values derived from proteomic analysis. “NA” indicates that no value was detected in that dataset.

In parallel, p53-mediated checkpoint pathways were activated following EAG1 loss, with p53 expression significantly increased, according to the RNAseq data (FC = 1.25, *p* = 0.0003). p53 functions as a natural safeguard during periods of rapid proliferation by halting cell cycle progression in response to oncogenic stress and is often downregulated in cancer, with over 50% of cancers carrying a loss-of-function mutation in the p53 gene (49, 50). The observed increase in p53 following EAG1 knockout suggests that EAG1 normally suppresses p53-dependent checkpoint signaling, thereby permitting rampant G1 progression. Loss of EAG1 appears to release this repression, enabling p53-mediated control mechanisms to restrain CDK–cyclin activity and enforce G1 arrest. Interestingly, MDM4 and MDM2, crucial proteins involved in the degradation of p53, were also upregulated in EAG1 knockout cells (MDM4: FC = 1.31, *p* = 0.00075, and MDM2: FC = 1.13, *p* = 0.0019), suggesting that EAG1 influences p53 signaling independently of the MDM2/MDM4 negative-feedback degradation pathway. Taken together, these findings align with the downregulation of the E2F pathway identified in our multi-omics analyses.

### Loss of EAG1 channel decreases TGF-β induced EMT

Our multi-omics results suggest that EAG1 ablation downregulates the EMT pathway. To further investigate the contribution of EAG1 channels in EMT, we employed TGF-β to induce EMT. TGF-β signaling alters the transcription of key EMT regulators through SMAD2/3 pathways, ultimately driving loss of epithelial polarity and stable cell-cell adhesion while promoting mesenchymal traits such as cytoskeletal plasticity, motility, and invasive capacity (51). Importantly, A549 cells are a well-established model for studying EMT due to their robust and reproducible response to TGF-β stimulation (52). EMT is associated with characteristic changes in cell morphology, transitioning from a compact “brick-like” epithelial shape to more “elongated” mesenchymal phenotype (53). To confirm that TGF-β treatment successfully induced ETM, we imaged WT and EAG1-KO cells cultured in the absence or presence of TGF-β. Image analysis revealed that both WT and EAG1-KO A549 cells underwent a robust EMT, characterized by a pronounced morphological shift toward an elongated, mesenchymal phenotype, whereas untreated cells retained their compact, epithelial “brick-like” morphology (Fig. 8*A*). Quantitative analysis revealed that the length of WT and EAG1-KO A549 cells was statistically similar in the absence of TGF-β, and both WT and EAG1-KO cells exhibited comparable elongation in response to TGF-β (Figure S5). Together, the imaging and quantitative analysis suggests that EAG1 knockout does not significantly alter A549 cell morphology either prior or following EMT induction.

**Figure 8:**
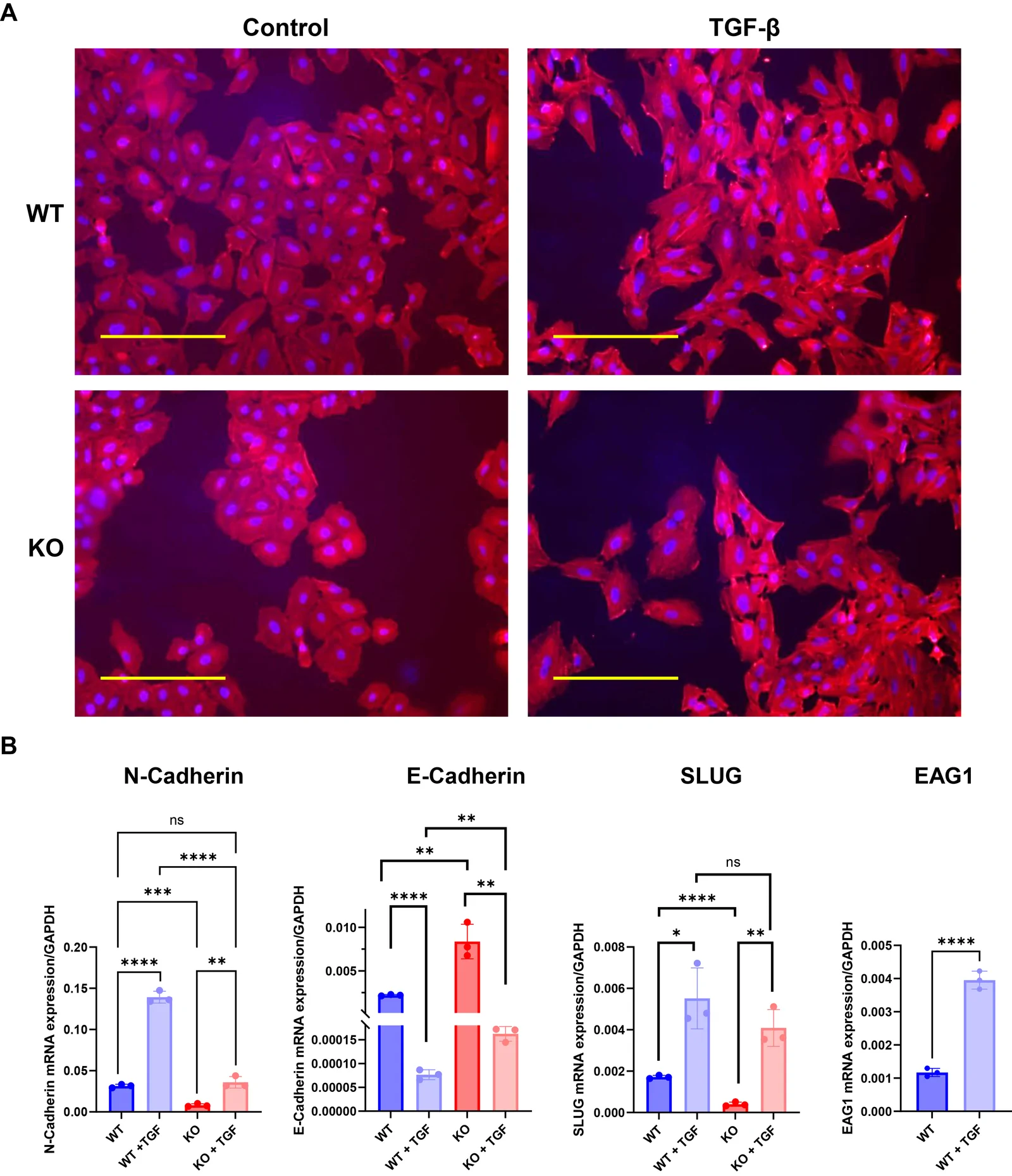
**TGF-β treatment induces EMT in A549/WT cells, while having a reduced effect in EAG1-KO cells.***A*, representative fluorescence images of A549/WT cells (*top*) and A549-EAG1-KO (*bottom*), either untreated (*left*) or treated with TGF-β (*right*) at 48 h after cell plating. Cells were stained for F-actin (phalloidin-Texas Red-X) and nuclear DNA (DAPI). Scale = 0.2 mm. *B*, bar graphs of mRNA expression of N-cadherin, E-cadherin, SLUG, and EAG1 relative to GAPDH expression for A549/WT cells (*blue*), A549/WT cells + TGF-β (*light blue*), A549/EAG1-KO cells (*red*), and A549/EAG1-KO cells + TGF-β (*light red*). Data represent three repeated experiments. The data are presented as mean ± SD. Unpaired Student’s *t* test analysis was used to determine statistical significance (∗∗∗∗*p* < 0.0001; ∗∗∗*p* < 0.001; ∗∗*p* < 0.01; ∗*p* < 0.05; ns *p* > 0.05). EAG1, Ether-à-go-go 1; EMT, epithelial–mesenchymal transition; TGF-β, transforming growth factor-beta.

It is well documented that morphological changes in EMT can be decoupled from changes in underlying cellular pathways (54, 55). To investigate the differences in EMT between WT and KO cells at the molecular level, we compared the expression of hallmark EMT markers, including E-cadherin, N-cadherin, and SLUG. RT-qPCR analysis further confirmed EMT induction in WT A549 cells, with significant upregulation of mesenchymal markers such as SLUG and N-cadherin and downregulation of the epithelial marker E-cadherin (Fig. 8*B*). Notably, successful EMT induction in WT cells was accompanied by an overall increase in EAG1 expression, consistent with the previous report (28). In contrast, EAG1-KO cells displayed markedly attenuated EMT responses following TGF-β treatment. In the absence of TGF-β, loss of EAG1 significantly reduced N-cadherin expression relative to WT cells, and TGF-β treatment was unable to fully rescue this deficit. Specifically, N-cadherin expression normalized to GAPDH increased to 0.139 ± 0.007 in WT cells following TGF-β treatment and only to 0.036 ± 0.007 in EAG1-KO A549 cells. These findings indicate that EAG1 is critical for the upregulation of N-cadherin during EMT. Concurrently, an opposing trend was observed for the epithelial marker E-cadherin, consistent with a reversal of the mesenchymal phenotype. EAG1 knockout significantly increased E-cadherin levels and TGF-β treatment only partially rescued this effect. Specifically, TGF-β reduced the normalized E-cadherin expression to 0.0000765 ± 0.00001 in WT cells and to 0.00016 ± 0.000016 in EAG1-KO cells. Interestingly, SLUG expression exhibited a different pattern. Loss of EAG1 significantly reduced SLUG expression in the absence of TGF-β. However, TGF-β treatment fully rescued this deficit by increasing normalized SLUG expression to 0.006 ± 0.00147 in WT cells and to 0.0041 ± 0.0009 in EAG1-KO cells, with no significant difference between expression levels in WT and EAG1-KO cells. Collectively, these data demonstrate that EAG1 plays a significant role in TGF-β–induced EMT, with a particularly strong influence on the cadherin switch. The ability of TGF-β to fully rescue SLUG expression suggests that EAG1 is not involved in the SLUG pathway during EMT signaling.

### EAG1 knockout inhibits A549 cell migration during EMT

Acquisition of an EMT phenotype is associated with an enhanced metastatic potential. Therefore, we next examined whether EAG1 influences EMT-driven cell migration. To address this, we performed wound-healing assays in WT and EAG1-KO A549 cells in the presence or absence of TGF-β (Fig. 9*A*). As expected, TGF-β treatment significantly increased migration in WT cells, with wound closure reaching on average 68.10 ± 15.6% in the presence of TGF-β and only 35.02 ± 11.27% in the absence (Fig. 9*B*). In contrast, EAG1-KO cells completely lacked EMT-induced migratory responses, displaying comparable wound healing in the absence and presence of TGF-β (16.39 ± 5.337% *versus* 21.59 ± 6.83%, respectively; Fig. 9*B*). Taken together, these findings are consistent with the RT-qPCR data and indicate that EAG1 channels are essential for EMT-associated migratory behavior.

**Figure 9:**
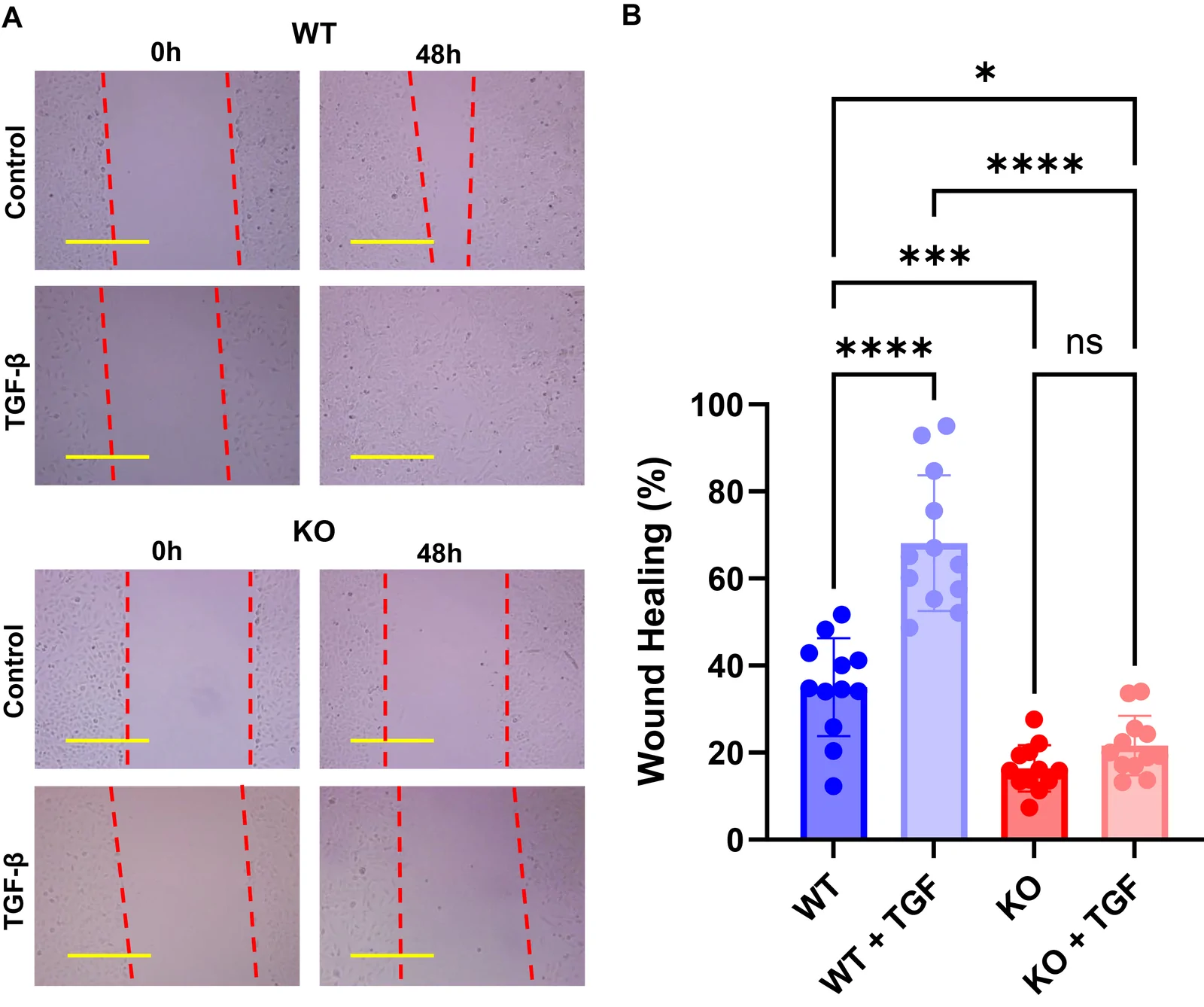
**Knocking out EAG1 decreases EMT-mediated migration.***A*, representative images of the wound healing of WT and EAG1-KO A549 cells untreated (control) and treated with TGF-β, as indicated, at 0 and 48 h after scratch. Scale bar = 0.5 mm. *B*, bar graph summary of the % wound healing of WT cells and EAG1-KO A549 cells in the absence or presence of TGF-β, as indicated, at 48 h postscratch. The data in the absence of TGF-β are the same as in Figure 5*A*. Data represent four repeated experiments, with each having three technical replicates for each cell line. The data are presented as mean ± SD. One-way ANOVA analysis was used to determine statistical significance (∗∗∗∗*p* < 0.0001; ∗∗∗*p* < 0.001; ∗*p* < 0.05; ns *p* > 0.05). EAG1, Ether-à-go-go 1; EMT, epithelial–mesenchymal transition; TGF-β, transforming growth factor-beta.

## Discussion

In this study, we demonstrate that the voltage-gated potassium channel EAG1 plays a central role in regulating tumor growth and metastatic progression in NSCLC A549 cells. Using a genetic ablation of EAG1 channels in A549 cells coupled with tissue culture approaches, zebrafish xenografts and multi-omics analysis, we show that loss of EAG1 impairs tumor growth both *in vitro* and *in vivo*, alters cell cycle progression, suppresses migratory and invasive behavior, and disrupts EMT. Together, these findings position EAG1 as a multifunctional regulator of proliferative signaling and metastatic ability in NSCLC.

Historically, a major bottleneck in investigating the exact contribution of EAG1 to tumorigenesis has been the lack of EAG1-knockout cell lines. Except a recent study using an EAG1-knockout MDA-MB-231 breast cancer cell line (29), prior research relied on manipulating EAG1 activity *via* either RNAi, nonspecific EAG1 inhibitors or EAG1-specific antibodies (2, 10, 30), each carrying experimental limitations (5, 31). RNAi provides only a temporary reduction in protein expression, which may wear off over the duration of an experiment. Consequently, RNAi-based approaches cannot capture the long-term effects of EAG1 ablation on chronic tumor progression and are poorly suited for long-lasting *in vivo* xenograft experiments. Furthermore, known EAG1 small-molecule inhibitors are nonspecific and typically concurrently inhibit hERG1 channels, which also play a role in cancer (5, 32, 33, 34). For example, while the EAG1 inhibitor astemizole is frequently used to study the role of EAG1 in cancer, its concurrent inhibition of hERG1 makes it difficult to clearly differentiate the effects of EAG1 inhibition from those of hERG1 inhibition. Finally, EAG1-specific antibodies decrease cancer growth *in vitro* and *in vivo* in mouse xenograft models. However, antibody accessibility and penetration into solid tumor tissues are common limitations for *in vivo* experiments, leaving the exact fraction of inhibited EAG1 channels unclear. By utilizing the CRISPR/Cas9 approach to achieve genetic silencing of EAG1 channels, our study successfully bypasses these historical limitations to reveal the full, long-term extent of EAG1-dependent effects in cancer biology.

Additionally, our study provides the first investigation of EAG1-dependent cellular pathways using integrated transcriptomic and proteomic approaches, generating an extensive omics dataset that will serve as an invaluable resource for future investigations of EAG1-dependent pathways. Finally, while prior studies indicate that EAG1-dependent cellular pathways are highly cancer-type specific (19, 24), its role in lung cancer has remained fundamentally unclear. The three prior publications related to EAG1 in lung cancer do not resolve whether EAG1 acts as a driver of tumorigenesis or merely an upregulated bystander. The first study found that EAG1 expression is upregulated during EMT in A549 lung cancer cells (28). However, because the nonspecific EAG1 inhibitor astemizole failed to prevent EMT, it remained unclear whether EAG1 was a driver, contributing *via* its nonconductive properties, or if the drug concentration was simply insufficient under the experimental conditions to elicit an effect. The study also left open a possibility that EAG1 is not a player in EMT at all but merely an upregulated bystander, an ambiguity compounded by the fact that the data for the astemizole experiments were not shown in the manuscript, making it impossible to assess if astemizole had some or no effect on EMT. The second study demonstrated that serum deprivation increases EAG1 expression *via* epidermal growth factor and ERK1/2 pathways; however, the study did not utilize any pharmacological or genetic modulation of EAG1 itself to pinpoint its downstream effects in lung cancer (23). The third study focused primarily on the therapeutic synergy of combining astemizole with gefitinib (an EGFR inhibitor), leaving the underlying molecular mechanisms unexamined (56).

Here, we directly address the deficit in EAG1 studies in cancer by using genetic silencing of EAG1 and omics to identify EAG1-dependent pathways in lung cancer. The dual-gRNA editing strategy employed in our study minimizes the possibility of off-target cuts. Additionally, the absence of compensatory changes in hERG1 expression (Fig. 1*E*) and the parallel transcriptomic and proteomics alignments (Fig. 7, *F* and *G*) strongly mitigate the likelihood of CRISPR-induced off-target effects. However, it is important to note that a formal genetic rescue of EAG1 in the knockout background was not evaluated in this study. Consequently, while our findings establish a clear functional importance for EAG1 in A549 cell-cycle progression and EMT, future work utilizing an EAG1 re-expression model will be valuable for mapping the exact steps responsible for restoring the proliferative and metastatic traits.

EAG1 channels can contribute to tumorigenesis *via* their canonical ion-conducting function and also *via* nonconductive properties most likely mediated by interactions with cellular scaffold (28, 57). The mechanisms identified in our study may reflect both ion-conductive and scaffolding functions of EAG1. The genetic modification used in our study, a 61-base-pair excision in the S2-S3 loop, is expected to introduce a premature stop codon, thereby preventing the expression of functional EAG1 channels. This is supported by our Western blot analysis using antibodies targeting the C-terminal region of EAG1, which indicates that the expression of the full-length EAG1 protein is significantly reduced in the EAG1-KO A549 cells, and functional analysis indicating that EAG1 currents are substantially reduced in EAG1-KO A549 cells compared to WT cells (Fig. 1). Furthermore, because the C-terminal region of EAG1 contains the essential assembly domain required for tetramerization (58), any truncated EAG1 protein containing the PAS, S1, and S2 segments that manages to bypasses cellular quality control systems will not form tetramers at the plasma membrane. Moreover, our RT-qPCR results indicate that the genetic modification substantially decreases EAG1 mRNA levels in the EAG1-KO cells. The decrease in transcript levels often is due to the nonsense-mediated mRNA decay following an exonic frameshift mutation (59, 60). However, this marked reduction is also highly consistent with the disruption of an autoregulatory pathway. Previous studies have showed that EAG1 expression is coupled with the upregulation of E2F transcription factors, which can directly bind to the EAG1 promoter to enhance EAG1 transcription (19, 61). Our global multi-omics analysis demonstrates that EAG1 knockout represses the E2F transcriptional machinery (Fig. 7). Therefore, EAG1 ablation likely dismantles the positive feedback loop, compounding the observed suppression of *KCNH1* transcript expression. Taken together, the genetic modification in the *KCNH1* gene diminishes both ion-conductive and nonconductive properties of EAG1 channels. Consequently, based on the current study alone, we cannot differentiate whether the EAG1-dependent effects observed in the EAG1-KO A549 cells stem from the loss of EAG1 ion permeation or the disruption of its interactions with the cellular scaffold, as the observed phenotypes may reflect the loss of both ion-conductive and scaffolding functions of EAG1.

Our flow cytometry and multi-omics results indicate that the reduction of proliferative ability of A549 cells due to EAG1 ablation is accompanied by a clear alteration in cell cycle progression. Flow cytometry analysis revealed an accumulation of EAG1-KO cells in G1 phase (Fig. 4), and transcriptomics analysis revealed strong transcriptional repression of transcripts for multiple CDKs and cyclins, with partial agreement at the protein level (Table 1). These findings align with the downregulation of the E2F pathway identified in our transcriptomic and proteomic analyses (Fig. 7), as graphically summarized in Figure 10*A*. E2F transcription factors are key drivers of G1/S transition, and suppression of E2F signaling provides a mechanistic explanation for the observed G1 arrest and reduced proliferation. Multiple studies have demonstrated a direct correlation between increased E2F signaling and elevated EAG1 expression (30, 61). Overexpression of pRb by transient transfection results in decreased EAG1 expression, while expression of the HPV E7 oncoprotein, which disrupts pRb–E2F complexes, leads to increased EAG1 expression (18, 30). It has been shown that E2F1 directly binds the EAG1 promoter, which leads to an increase in EAG1 expression (19, 61). Although a consensus in the field is that EAG1 is an important player in cell cycle regulation, there is some disagreement in the findings and also the effect might be cancer-type specific. For instance, knocking down EAG1 in cervical cancer HeLa cells led to G2/M arrest, while in a gastric cancer cells G1-arrest (19, 24). Taken together with our findings of G1-phase arrest in EAG1-KO A549 cells, these studies suggest that EAG1-dependent cell cycle regulation is cancer-type specific, underscoring the importance of studying the effect in different cancer types.

**Figure 10:**
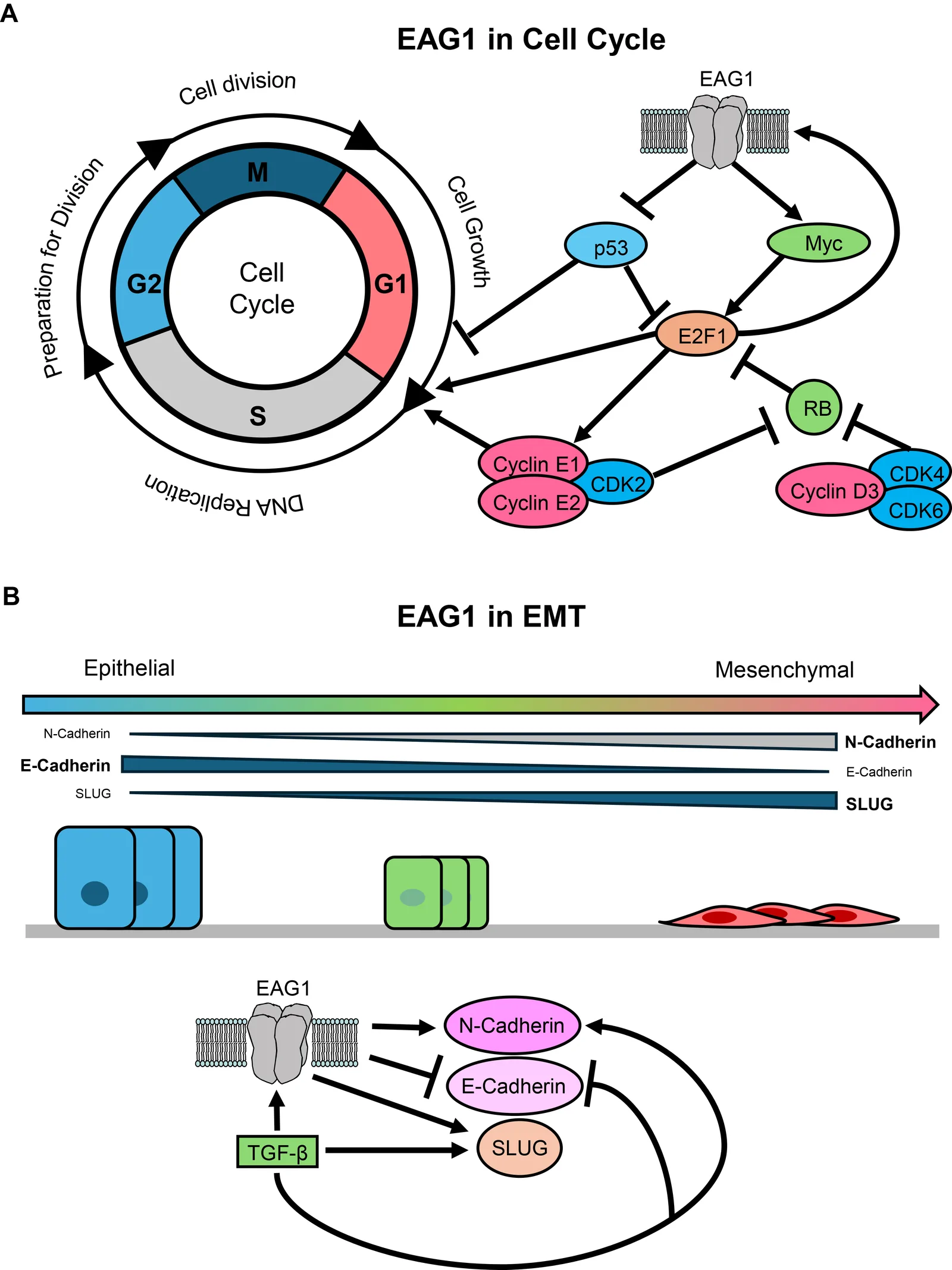
**EAG1 in cell cycle and EMT in A549 cells.***A,B,* graphic summary of the EAG1-associated regulation in cell cycle (*A*) and EMT (*B*) in A549 cells uncovered by our experiments. The connections depicting EAG1-dependent inhibition or activation may not be direct and may contain intermediate players that have yet to be uncovered. EAG1, Ether-à-go-go 1; EMT, epithelial–mesenchymal transition; TGF-β, transforming growth factor-beta.

Beyond its role in cell growth, our results demonstrate that EAG1 is a regulator of metastatic traits of NSCLC. Loss of EAG1 significantly reduced migration and invasion *in vitro*, as shown by impaired wound closure and decreased endothelial barrier penetration (Fig. 5). These phenotypes translated to the *in vivo* settings, where EAG1 knockout markedly reduced extravasation in zebrafish xenografts and decreased the aggressiveness of intravasation events (Figs. 6 and S4), suggesting that EAG1 may be especially important for late-stage metastatic dissemination, a step that is often rate-limiting in the metastatic cascade (62). Furthermore, our results indicate that EAG1 plays an important role in EMT, a crucial process for metastasis (63). A link between EAG1 and EMT has been reported before in the study by Restrepo-Angulo *et al.*, where it was shown that EAG1 protein expression is enhanced during EMT in A549 cells (28). However, the direct involvement of EAG1 channels and their contribution to EMT was not examined in the study. Our *in vitro* experiments and integrated multi-omics analyses identified EMT as one of the most significantly downregulated pathways upon EAG1 knockout. Functionally, EAG1-KO cells exhibited a blunted EMT response to TGF-β, characterized by selective disruption of the cadherin switch (Fig. 8*B*). In particular, the loss of EAG1 severely limited N-cadherin expression during EMT, a hallmark of mesenchymal phenotype and a critical mediator of invasive behavior. A similar trend was observed for E-cadherin, where TGF-β was able to repress the expression, although not to the level of WT cells, demonstrating a partial rescue. In contrast, SLUG induction was fully rescued by TGF-β, indicating that EAG1 regulation in EMT pathway is specifically focused on the cadherin switch pathway, as graphically summarized in Fig. 10*B*. The critical role of EAG1 in EMT was further corroborated with an integrated transcriptomic and proteomic analysis. GSEA identified the EMT pathway as one of the most robustly and significantly downregulated networks upon EAG1 ablation, yielding a highly significant NES of −2.07 and FDR of 0.00. Crucially, this broad suppression of the EMT program was not restricted to transcription as our proteomic profiles strongly converged with the transcriptomic landscape, demonstrating a highly significant correlation across EMT-associated targets (Fig. 7, *E* and *G*). This global, multilayered suppression across EMT-related transcripts and proteins demonstrates that the core EMT machinery is fundamentally altered in the absence of EAG1.

It has been shown that migration in many cancer cell types depends on membrane hyperpolarization regulated by potassium channels, including EAG1 (27, 64, 65). Increased K^+^ efflux hyperpolarizes the membrane and increases the driving force for Ca^2+^ entry through calcium channels, thereby increasing the concentration of intracellular Ca^2+^—a major regulator of many oncogenic processes (66, 67, 68). In a breast cancer cell line, it was shown that EAG1 enhances Ca^2+^ entry *via* Orai1, a key component of store-operated Ca^2+^ channels, in order to regulate cell migration but not cell proliferation (27). Consistent with these findings, a recent study from our research group demonstrated that knocking out EAG1 in MDA-MB-231 breast cancer cells significantly decreases cell migration but has no statistically significant effect on cell growth (29). The lack of effect of EAG1-dependent Orai1 regulation on the proliferation of breast cancer cells is in disagreement with results in cervical cancer and osteoblast cells, where it was shown that Orai1 channels control cell proliferation, by modulating G1 cell-cycle regulators, such as cyclin D1, cyclin E, CDK4, and CDK6 (69, 70). This disagreement could indicate a tumor-specific effect of Orai1 channels. Overall, the role of EAG1 in regulating membrane potential and the Ca^2+^ homoeostasis through Orai1 channel could explain some of the findings on metastasis and proliferation seen in this study. However, this represents only part of the oncogenic mechanism, as EAG1’s tumorigenic effects are not solely dependent on its function as an ion channel. It has been previously shown that abolishing ion permeation through EAG1 *via* a pore-disrupting point mutation does not fully prevent EAG1-dependent malignant transformation of NIH/3T3 mouse embryonic fibroblasts, indicating that EAG1 channels can contribute to tumorigenesis through an ion conduction-independent (non-canonical) pathway (57). Unraveling the precise, step-by-step biochemical relay connecting EAG1 channel activity at the cell-surface to the downstream signaling, including E2F activation, pRb control, or specific EMT transcriptional machinery complexes, represents a critical direction for future investigation.

A549 cells harbor a constitutively active, homozygous KRAS mutation G12S common in NSCLC (71). The KRAS pathway plays an important role in NSCLC by serving as a primary intracellular signaling hub that orchestrates the downstream MAPK/ERK and PI3K/AKT cascades, driving uncontrolled cell proliferation, metabolic reprogramming, and survival (72). Our GSEA transcriptomic profiling revealed a complex modulation of KRAS-associated pathways following EAG1 ablation, characterized by the positive enrichment of both the KRAS_SIGNALING_UP (genes typically upregulated by KRAS activation) and KRAS_SIGNALING_DN (genes typically suppressed by KRAS activation) hallmark sets (Fig. 7*C*). These transcriptomic results suggest a complex pathway rewiring under genetic stress caused by the ablation of EAG1. The ultimate indication of whether the gene expression shift is protumorigenic or antitumorigenic lies in the physical phenotype of the EAG1-KO cells. Throughout our entire study, our functional results consistently demonstrate that EAG1-KO robustly suppresses cell proliferation, metabolic viability, and *in vivo* tumor progression. Taken together, our results demonstrate that despite the aggressive baseline context of a constitutively active KRAS driver mutation in A549 cells, genetic ablation of EAG1 effectively compromises the tumor's core oncogenic drive, positioning this channel as a viable therapeutic target in NSCLC.

It is important to acknowledge some limitations of our study. The genetic modification used in our study introduces a premature stop codon in the S2-S3 loop. It is expected that the truncated EAG1 protein containing the PAS, S1, and S2 segments will be efficiently degraded by cellular quality control mechanisms (73, 74, 75). However, we cannot exclude the possibility that some truncated proteins will bypass cellular quality control systems. While these proteins would be unable to form functional channels and would be deficient in scaffolding interactions, they might still contribute to the observed cellular phenotypes. Although zebrafish xenografts provide compelling *in vivo* evidence that EAG1 loss reduces tumor growth, migration, and extravasation, confirming these findings in mammalian systems is a critical next step. Our study is focused solely on A549 cells. We selected A549 cells because they are widely used in lung cancer research and are considered a gold standard for NSCLC models. However, extending our findings into additional NSCLC cell lines with varying genetic backgrounds will further establish the clinical importance of EAG1 in lung cancer. Therefore, future studies should investigate the functional contribution of EAG1 channels across a broader panel of NSCLC cell lines. Finally, it is important to emphasize that while A549 cells provide an excellent model for isolating the fundamental EAG1-dependent biological mechanisms in NSCLC, they lack the complex architecture, microenvironmental stromal interactions, and patient-specific genetic heterogeneity inherent to primary cancer tissues. Consequently, future validation in primary patient tumors and clinical cohorts remains a necessary step to confirm the translational relevance of our findings to tumor progression in patients.

In summary, our findings support a unified model in which EAG1 is a major driver of tumor progression in A549 cells by regulating proliferative signaling (*via* E2F and cell cycle control) and EMT-dependent migratory and metastatic pathways. This dual role is particularly relevant in NSCLC, where tumor growth and early metastatic dissemination often occur concurrently (76). Therefore, our study establishes EAG1 as a central regulator of A549 cell growth and metastasis, providing a strong rationale for targeting EAG1-mediated pathways for lung cancer treatment.

## Experimental procedures

### Cell culture

Wildtype (WT) A549 cells (CCL-185) were purchased from the Tissue Culture and Biobanking Shared Resource at Georgetown University. A549 cell line derived from a single cell clone with knocked out EAG1 (EAG1-KO) was generated by Ubigene using CRISPR/Cas9 technology (YKO-H1554). The identities of the WT and EAG1-KO cell lines were validated *via* short tandem repeat profiling using the PowerPlex 16HS PCR kit (Promega) at the Tissue Culture and Biobanking Shared Resource at Georgetown University Medical Center. Both cell lines matched the A549 reference profile (CVCL_0023), and no cross-contamination was detected. The short tandem repeat results were analyzed using GeneMarker software, version 1.85 (SoftGenetics). The corresponding allele table and electropherograms are shown in Figure S6. Both WT and EAG1-KO cell lines were cultured in Ham’s F12K- media (Gibco) supplemented with 10% (v/v) heat-inactivated FBS and 1% (v/v) penicillin/streptomycin (Gibco). Trypsin-free PBS-EDTA was used for cell detachment from the tissue culture dishes to avoid potential cleavage of EAG1 channels. Cells were cultured at 37 °C and 5% CO2. To induce EMT, 50,000 WT and EAG1-KO cells were seeded into 6-well plates. After 24 h, the wells were treated with culture media either supplemented with or without 5 ng/ml of TGF-β (Gibco).

### Polymerase chain reaction experiments

Total RNA was extracted from the tested cells using the RNeasy Mini Kit (Qiagen GmbH). RNA concentration and purity were assessed by Nanophotometer P330 (Implen). First-strand cDNA was synthesized from 1 μg of total RNA using random hexamer primers and a commercial reverse transcription kit (High-Capacity cDNA Reverse Transcription Kit from Applied Biosystems). The resulting cDNA was diluted to a working concentration of 10 ng/μl. The PCR was run with 1 μg of cDNA, Tag polymerase buffer (BioLabs, B9014S), dNTPs (BioLabs, U151B), Tag DNA polymerase (BioLabs, M0273X), and run on T100 Thermal Cycler (Bio-Rad). The PCR products were run on 1% agarose gel at 120V for 30 min and visualized with SyberSafe DNA Gel Stain (Invitrogen, S33102) using Gel Doc EZ Imager (Bio-Rad). Primer sequences for EAG1 were as follows: Forward 1 (F1): 5′-CCTGACGATTTATCCAGAGTATTG-3′, Forward 2 (F2): 5′-GTGATTTCTGACCCCAAACTTATC-3′, and Reverse 1 (R1): 5′-GGCCTCCAAACCGCCAAAGG-3′.

### Reverse transcription-quantitative polymerase chain reaction experiments

EAG1 mRNA levels were quantified using the RT-qPCR. Total RNA was extracted from the tested cells using the RNeasy Mini Kit (Qiagen GmbH). RNA concentration and purity were assessed by Nanophotometer P330 (Implen). First-strand cDNA was synthesized from 1 μg of total RNA using random hexamer primers and a commercial reverse transcription kit (High-Capacity cDNA Reverse Transcription Kit from Applied Biosystems). The resulting cDNA was diluted to a working concentration of 10 ng/μl. RT-qPCR was performed using the CFX Connect Real-Time PCR Detection System (Bio-Rad) and SYBR Green chemistry. Each 25 μl reaction contained 12.5 μl iQ SYBR Green Supermix (Bio-Rad), 2 μl cDNA template, 1 μl of forward and reverse primers each at 10 μM concentration, and nuclease-free water to volume. The thermal cycling protocol consisted of an initial denaturation at 95 °C for 3 min, followed by 40 amplification cycles (95 °C for 15s, 57.5 °C for 1 min). Forward and reverse primer sequences for EAG1 were 5′-TCTGTCCTGTTTGCCATATGATGT-3′ and 5′-CGGAGCAGCCGGACAA-3′, respectively. Forward and reverse primer sequences for hERG1 were 5′-ACGTCTCTCCCAACACCAAC-′3 and 5′-GAGTACAGCCGCTGGATGAT-3′, respectively. Forward and reverse primer sequences for N-cadherin were 5′-CCTTTCAAACACAGCCACGG-′3 and 5′-TGTTTGGGTCGGTCTGGATG-3′, respectively. Forward and reverse primer sequences for SLUG were 5′-CCCTCACTGCAACAGAGCAT-′3 and 5′-TACACAGCAGCCAGATTCCTC-3′, respectively. Forward and reverse primer sequences for E-cadherin were 5′-TGCACCAACCCTCATGAGTG-′3 and 5′-GTCAGTATCAGCCGCTTTCAG-3′, respectively. GAPDH was used as the internal reference gene. Forward and reverse primer sequences for GAPDH were 5′-GGAGCGAGATCCCTCCAAAAT-3′ and 5′-GGCTGTTGTCATACTTCTCATGG-3′, respectively. Fluorescence signal was detected *via* SYBR Green intercalation. Each sample was run in triplicate, and nontemplate controls were included to monitor for contamination. Relative gene expression levels were quantified by normalizing to GAPDH. Unpaired Student’s *t* test was performed to analyze differences in gene expression. *p* < 0.05 was considered statistically significant.

### Western blotting

Cells were lysed with PierceTM RIPA buffer (Thermo Fisher Scientific) supplemented with protease inhibitors (c0mplete mini, protease inhibitor cocktail, Roche) according to the manufacturer’s protocol. Proteins were run through a precast gel (Invitrogen, NuPAGE 12% Bis-Tris Gel) at 150V for 60 min. Dry protein membrane transfer was performed using the iBlot 2 Dry Blotting System (Invitrogen). Proteins were stained using anti-EAG1 antibody (Alomone Labs Cat# APC-104) (1:500 dilution), and anti-actin antibody (Abcam Cat# ab6276) (1:2000 dilution). Each sample was split between two gels. One blot was probed with anti-EAG1 antibodies and the other with anti-actin antibodies. Stained proteins were detected using SuperSignal West Femto Maximum Sensitivity Substrate kit (Thermo Scientific, 34095) on the Odyssey FC imager (Li-Cor). Band intensities were quantified using ImageJ. Background signal was subtracted for each band, and the intensity of each EAG1 band was normalized to its corresponding actin loading control from the same sample.

### Microscopy

To assess cells growth, 50,000 WT or EAG1-KO A549 cells were seeded into 6-well plates. Three representative images were taken following 24 h and 72 h of culturing using the Olympus IX-71 inverted microscope. To calculate changes in cell count, the cells were manually counted for each image using ImageJ. Changes in cell count were calculated for each well individually by normalizing to the 24-h time point. The experiments were repeated three times. Unpaired Student’s *t* test was used to analyze differences in the normalized cell count. *p* < 0.05 was considered statistically significant.

To visualize morphological changes following EMT induction, 50,000 WT or EAG1-KO A549 cells were seeded onto coverslips and cultured for 48 h in the absence or presence of 5 ng/ml TGF-β. Cells were fixed with 3.7% formaldehyde for 15 min, permeabilized with 0.5% Triton X-100 for 15 min, blocked with 5% bovine serum albumin for 1 h, and treated with Image-iT FX signal enhancer (Invitrogen #I36933) for 30 min, as previously described (77). Cells were stained with 0.25× Texas Red-X Phalloidin (Thermo Fisher Scientific, #T7471) for 1 h, followed by counterstaining with 300 nM 4′,6-diamidino-2-phenylindole for 10 min. Coverslips were mounted onto glass slides using ProLong Diamond Antifade Mountant (Thermo Fisher Scientific, #P36965). Images were collected using an Olympus IX-71 inverted fluorescence microscope. Cell length was analyzed using ImageJ software by manually measuring the length, defined as the longest cell dimension, of 50 different cells with clearly defined borders for each of the four experimental groups: WT and EAG1-KO cells cultured in the absence or presence of TGF-β.

### Cell viability

To compare the growth rate of WT and EAG1-KO A549 cells, the cells were plated at a density of 2000 cells per well into three 96-well plates, with each plate containing nine wells with WT and nine wells with EAG1-KO cells. Viability of cells for one of the plates was measured 24 h after plating, for another plate 48 h after plating, and for the last plate 72 h after plating. CellTiter-Blue assay (Promega) was used to determine cell viability. The CellTiter-Blue assay is based on the conversion of nonfluorescent resazurin into fluorescent resorufin by living cells. The resazurin fluorescence upon excitation at 560 nm was recorded at 590 nm with the Synergy H4 Plate Reader. To calculate the ratio of viable cells between WT and EAG1-KO cells, for each experimental day, the data were normalized to the fluorescence signal of the WT cells. Unpaired Student’s *t* test was performed to analyze differences in cell viability for each individual day. *p* < 0.05 was considered statistically significant.

### Real-time analysis of cell growth and invasion

Cell proliferation and invasion rates were measured continuously using an xCELLigence Real-Time Cell Analyzer Dual Purpose (RTCA DP) instrument (Agilent Technologies). To measure cell proliferation, WT and EAG1-KO A549 cells were collected from tissue-culture flasks using PBS-EDTA, and 10,000 cells per well were seeded into an E-Plate 16 PET plate (Agilent, #300600890) in the tissue culture media (78). CI measurements were recorded every 10 min until the CI curve reached a plateau for both cell lines. The experiment was repeated three times with three technical replicates for each, and statistical analyses were performed using a two-way ANOVA.

To study cell invasion, 25,000 HUVECs per well were seeded into an E-Plate 16 PET plate in HUVEC medium (Lonza, #CC-3162) (79). CI measurements were recorded every 3 min until the HUVEC cell monolayer became confluent, as represented by a stabilized CI curve reaching a plateau, which took approximately 24 h. At this point, the experiment was paused, and 10,000 WT or EAG1-KO A549 cells were added to the wells in two technical replicates. For the HUVEC-only control, only media were added. Changes in CI were then monitored every 3 min for 3 h. The experiment was repeated three times with two technical replicates. Statistical analysis was performed using an unpaired *t* test.

### Wound healing assay

For the wound healing assay, WT and EAG1-KO A549 cells were seeded at a density of 50,000 cells per well into 24-well plates. Once the cells reached 80% to 90% confluency, a wound was introduced by scratching across the bottom of the well with a 200 μl pipette tip.

Following the wound introduction, the cells were washed three times with PBS to remove the debris and were treated with either media alone (control group) or media containing 5 ng/ml TGF-β (treatment group). Both groups were in serum-deprived condition (1% FBS) to reduce the proliferation. For each well, three representative images were collected along the wound at 0 and 48 h after the wound introduction using an Olympus IX-71 inverted microscope. The wound closure (%) was calculated using the following formula:

$$Wound\;Closure\;\left(\%\right)=\frac{{Area}_{o}\;-\;{Area}_{t}}{{Area}_{0}}\;\times \;100$$

where, Area_0_ is the wound area at 0 h, while Area_t_ is the wound area at a specific measurement time (*e.g.*, 48 h). The wound area was calculated using the ImageJ Wound Healing Size Tool plugin. The experiments were repeated three times with three technical replicates. Changes from 0 h to 48 h were calculated for each well individually, and a comparison between the treatment and control groups was performed using two-way ANOVA. *p* < 0.05 was considered statistically significant.

### Electrophysiology and data analysis

For electrophysiology experiments WT or EAG1-KO A549 cells were grown on glass coverslips for 2 days until 50-70 confluency was reached. Currents from WT and EAG1-KO A549 cells were recorded using Axopatch 200B patch-clamp amplifier (Molecular Devices) and pClamp10 software (Molecular Devices). The signals were digitized using Digidata 1550 (Molecular Devices). Patch pipettes were pulled from borosilicate glass and had resistances of 1.2 to 2.5 MΩ after fire polishing. The extracellular (bath) solution contained 115 mM NaCl, 50 mM KCl, 1 mM CaCl_2_ꞏ2H_2_O, 10 mM Hepes, pH 7.4, adjusted with NaOH. Pipette solution contained 155 mM KCl, EDTA 5 mM, 10 mM Hepes, pH 7.3, adjusted with KOH. The EAG1 currents were elicited by applying a series of 1 s voltage pulses (ranging from −100 to +80 mV in 20-mV increments) from a holding potential of −80 mV, followed by a 1.5 s voltage pulse to −100 mV. Experiments were performed at room temperature.

### Zebrafish xenografts

All animal procedures were conducted in accordance with NIH guidelines for the care and use of laboratory animals and approved by the Georgetown University Institutional Animal Care and Use Committee (Protocol 2017-0078).

WT and EAG1-KO A549 cells were stained with the red fluorescence dye Vybrant DiI (Thermo Fisher, V22885), according to the manufacturer's protocol. For the comparison of growth rate, 2 dpf zebrafish embryos were microinjected into the yolk sac with 5 nl of cell suspension (4 × 10^7^ cells/ml), as described before (29, 80, 81, 82, 83). The zebrafish line *Tg(kdrl:GRCFP)zn1*, which expresses fluorescent protein GFP in the endothelial cells, was used. Around 100 embryos were injected for each cell line. Changes in tumor sizes were monitored daily for 3 days postinjection using an Olympus IX-71 inverted microscope, as previously described (80, 84). Tumor sizes were calculated using ImageJ by measuring tumor area (80). Animals lacking measurable tumors were excluded from the study. Changes from day 0 to day 3 were calculated for each individual fish, and comparisons between groups were performed using the unpaired Student’s *t* test.

For testing differences in extravasation, the DiI-stained cells (5 nl of 4 × 10^7^ cells/ml cell suspension) were injected directly into the circulation *via* the Duct of Cuvier, as previously described (84). Animals lacking tumor cells in the tail 1 h post-injection were removed. After 48 h, the tail region of each fish was imaged using the Olympus IX-71 inverted microscope, and cells outside of the vasculature were manually counted. Prior to analyses, the images were randomized, and only cells from the end of the intestinal duct to the tail were considered. A fish was considered to have extravasation if at least one cell was outside of the vasculature. Pearson's Chi-squared test was used to calculate statistical differences between extravasation for WT and EAG1-KO cells. *p* < 0.05 was considered statistically significant.

For testing differences in intravasation, the DiI-stained cells (5 nl of 4 × 10^7^ cells/ml cell suspension) were injected into precardiac space as previously described (84). Animals with tumor cells in the tail 1 h postinjection were removed. After 48 h, the tail region of each fish was imaged using the Olympus IX-71 inverted microscope, and cells in the tail area were manually counted. Prior to analyses, the images were randomized, and only cells from the end of the intestinal duct to the tail were considered. A fish was considered to have intravasation if at least one cell was in the tail of the fish. Pearson's Chi-squared test was used to calculate statistical differences between intravasation for WT and EAG1-KO cells. *p* < 0.05 was considered statistically significant.

Hypothermia, immersion in ice water, was used for euthanasia at the end of experiments. This is consistent with the recommendations of the panel on euthanasia of American Veterinary Medical Association. Sex of zebrafish embryos becomes clear only at the age of 3 months, well after the first week to which we limit our experiments. Therefore, sex as a biological variable does not need to be considered for the xenograft experiments described in this study.

### Flow cytometry

A549/WT- and EAG1-KO cells were incubated for 30h to a confluency of 50 to 70%. The cells were collected and washed three times in cold PBS. 1,000,000 cells were fixed in 1 ml 75% cold ethanol and stored at −20 °C. Cells were washed once in cold PBS and then stained with 1 ml of PI solution (PI solution: PBS containing 0.1% Triton X-100 with 50ug/ml RNase A and 50 ug/ml propidium iodide). Cells were incubated in dark at room temperature for 30 min and refrigerated until they were run. Samples were run the same day as staining. Cell cycle modeling was performed using Modfit (Verity Software). The experiment was repeated three times.

### RNA sequencing

Cells were harvested after 4 days of incubation when the confluency was 80 to 85%. Total RNA was extracted from WT and EAG1-KO A549 cells using the RNeasy Mini Kit (Qiagen GmbH). RNA concentration and purity were assessed by Nanophotometer P330 (Implen). Sequencing was performed by Novogene Inc. on a NovaSeq X Plus platform (PE150) averaging 40 million reads per sample. FASTQ files were quality-checked using FastQC (Babraham Institute), and reads were aligned to the human genome (hg38) with a mapping rate of at least 85.7% and quantified using the Salmon 1.10.0 tool (85). Differential gene expression analysis was conducted in RStudio with the edgeR 4.6.3 package (86), and GSEA (Broad institute) was performed using MSigDB Hallmark gene sets. NES was used to compare analysis across different gene sets, while FDR was used to assess significance. RNA sequencing was repeated three times.

### Proteomics

5,000,000 cells were lysed with 1 ml of PierceTM RIPA buffer (Thermo Scientific) supplemented with protease inhibitors (c0mplete mini protease inhibitor cocktail, Roche), according to the manufacturer’s protocol. Protein concentration in the cell lysates was determined with BCA assay, and equal amounts of proteins from each sample were processed for proteomics experiments, following a procedure published previously (87). In brief, samples were resuspended with 50 μl of 5% SDS buffer (containing 50 mM triethylammonium bicarbonate and 20 mM DTT) and heated for 10 min at 95 °C. After cooling down to room temperature, 4 μl of 500 mM iodoacetamide in 5% SDS solution was added to a final concentration of 40 mM and incubated in the dark for 30 min. The samples were centrifuged for 8 min at 13,000*g* to separate the undissolved matter. The supernatant was saved and loaded onto S-Trap column (ProtiFi, LLC). Proteins were digested with sequencing-grade Lys-C/trypsin (Promega) by incubation at 37 °C overnight. The resulting peptides were eluted and dried down with a SpeedVac (Fisher Scientific).

Peptides were analyzed with a reversed phase nanoElute 2 nanoflow liquid chromatography system (Bruker Daltonics Gmbh) coupled with a mass spectrometer timsTOF Ultra II (Bruker Daltonics Gmbh). In brief, samples in 0.1% formic acid solution were loaded and separated with an analytical column (PepSep C18 packed with ReproSil AQ as stationary phase, pore size 120 Å, 15 cm × 75 μm, 1.5 μm) with the temperature controlled at 50 °C. The flow rate was set as 300 nl/min. A 40-min gradient of buffer A (0.1% formic acid) and buffer B (0.1% formic acid in ACN) was used for separation: 2% buffer B at 0 min, 23% buffer B at 28 min, 30% buffer B at 30 min, 90% buffer B at 36 min, and 90% buffer B at 40 min. The CaptiveSpray source was used, with the ion spray voltage 1600 V. All data were acquired by the timsTOF Ultra mass spectrometer *via* Bruker timsControl in diaPASEF mode. Full mass spectrometry data were acquired in a range of 100 to 1700 m/z and 1.45 1/K0 [V-s/cm2] to 0.64 1/K0 [V-s/cm2] in diaPASEF. Data-independent acquisition windows ranged from 400 m/z to 1000 m/z and were acquired with ramp times of 100 ms or otherwise indicated. High sensitivity detection for low sample amounts was enabled without diaPASEF data denoising. The experiments were repeated three times.

Analysis of diaPASEF raw files was done by using Spectronaut (Biognosys, v20) in directDIA mode with default settings. In brief, dynamic retention time prediction with local regression calibration was selected. Interference correction on MS and MS2 level was enabled. The Qvalue cutoff was set to 1% at the peptide precursor and protein level using scrambled decoy generation and dynamic size at 0.1 fraction of library size. MS2-based quantification by area was used, enabling local cross-run normalization.

## Data availability

All data are contained in the article or available on request by contacting the corresponding author: tib5@georgetown.edu.

## Supporting information

This article contains supporting information.

## Conflict of interest

The authors declare that they have no conflicts of interest with the contents of this article.
