# Supplementary material for: EAG1 channel is a central regulator of tumor growth and metastasis in A549 non-small cell lung cancer cells
Source: J Biol Chem. 2026 Sep 23;302(11):113590. doi: 10.1016/j.jbc.2026.113590 (PMC13720809; doi:10.1016/j.jbc.2026.113590)
Supplement: Supplementary file 1 [file mmc4.pdf]

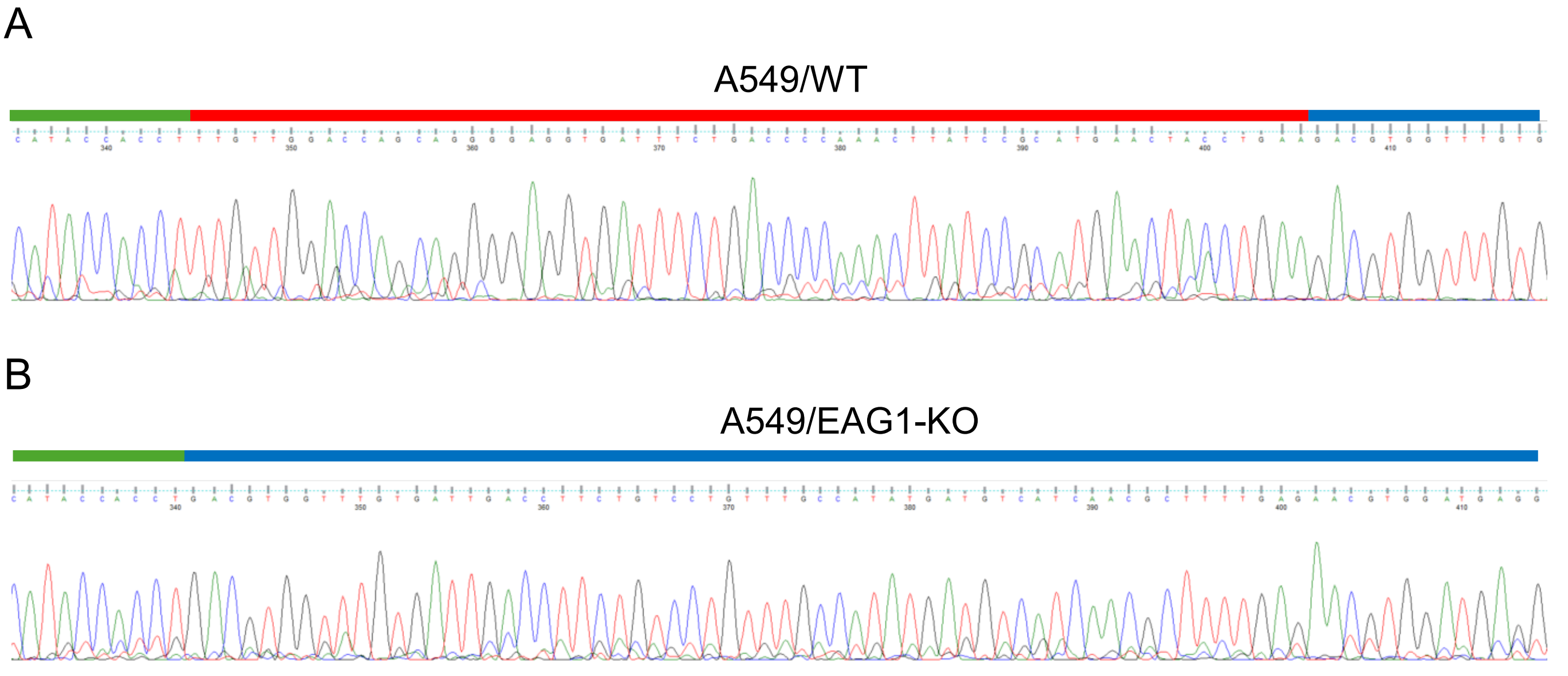

Supplementary Fig. 1. Validation of EAG1 gene knockout by Sanger sequencing. Chromatograms of the EAG1 gene locus from WT (A) and EAG1-KO (B) A549 cells. In the WT sequence, the 61-nucleotide region targeted for deletion is highlighted in red, with the upstream and downstream sequences shown in green and blue, respectively. In EAG1-KO cells, this 61-bp region is completely excised. Nucleotide trace peaks are color-coded as follows: adenine (A, green), guanine (G, black), cytosine (C, blue), and thymine (T, red).

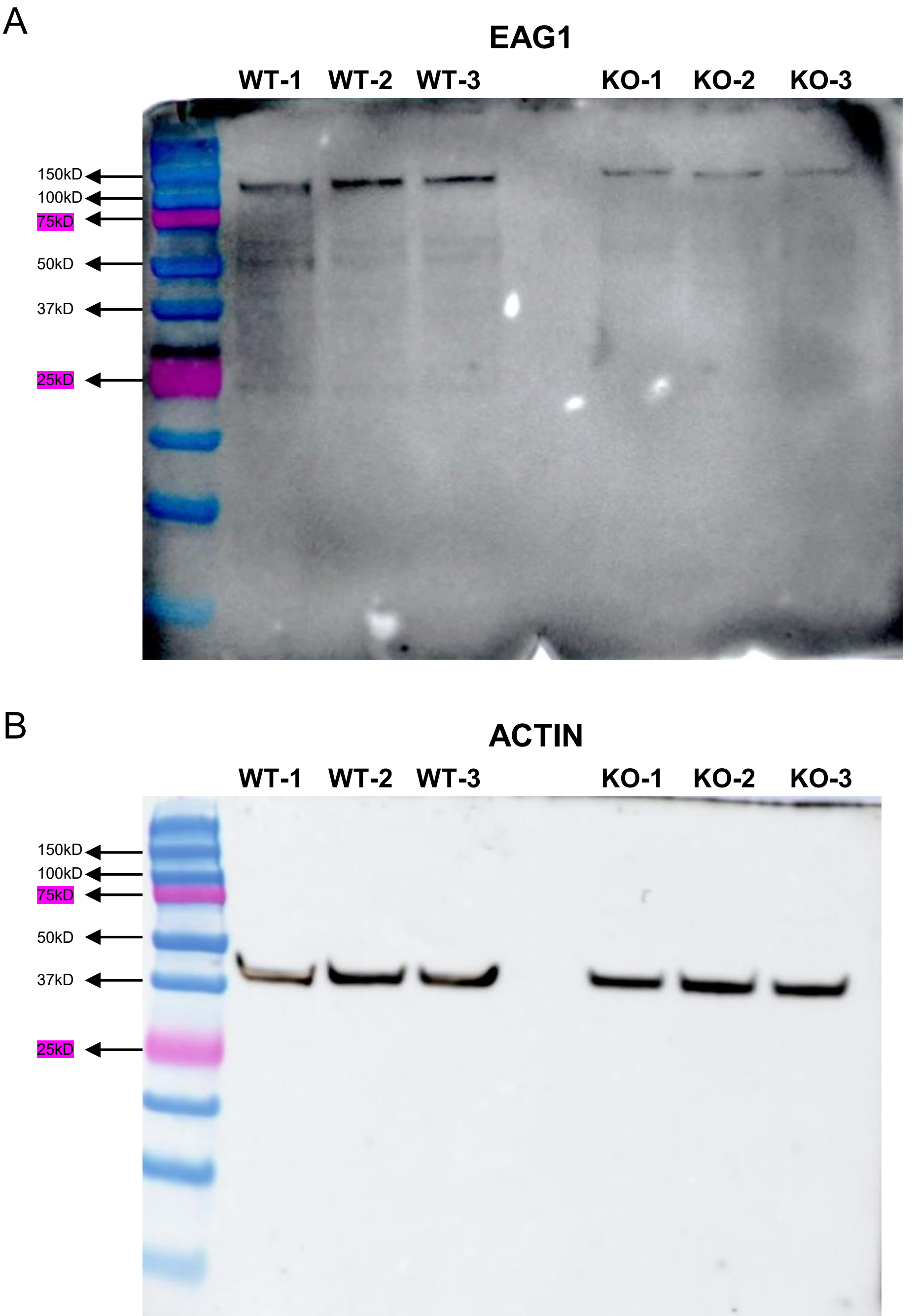

Supplementary Fig. 2. Validation of EAG1 protein knockout by Western blotting. Full-length, uncropped membrane images from three repeated experiments probed with anti-EAG1 (A) and anti-β-actin (B) antibodies. WT-3 and KO-1 were selected as representative images for Fig. 1C.

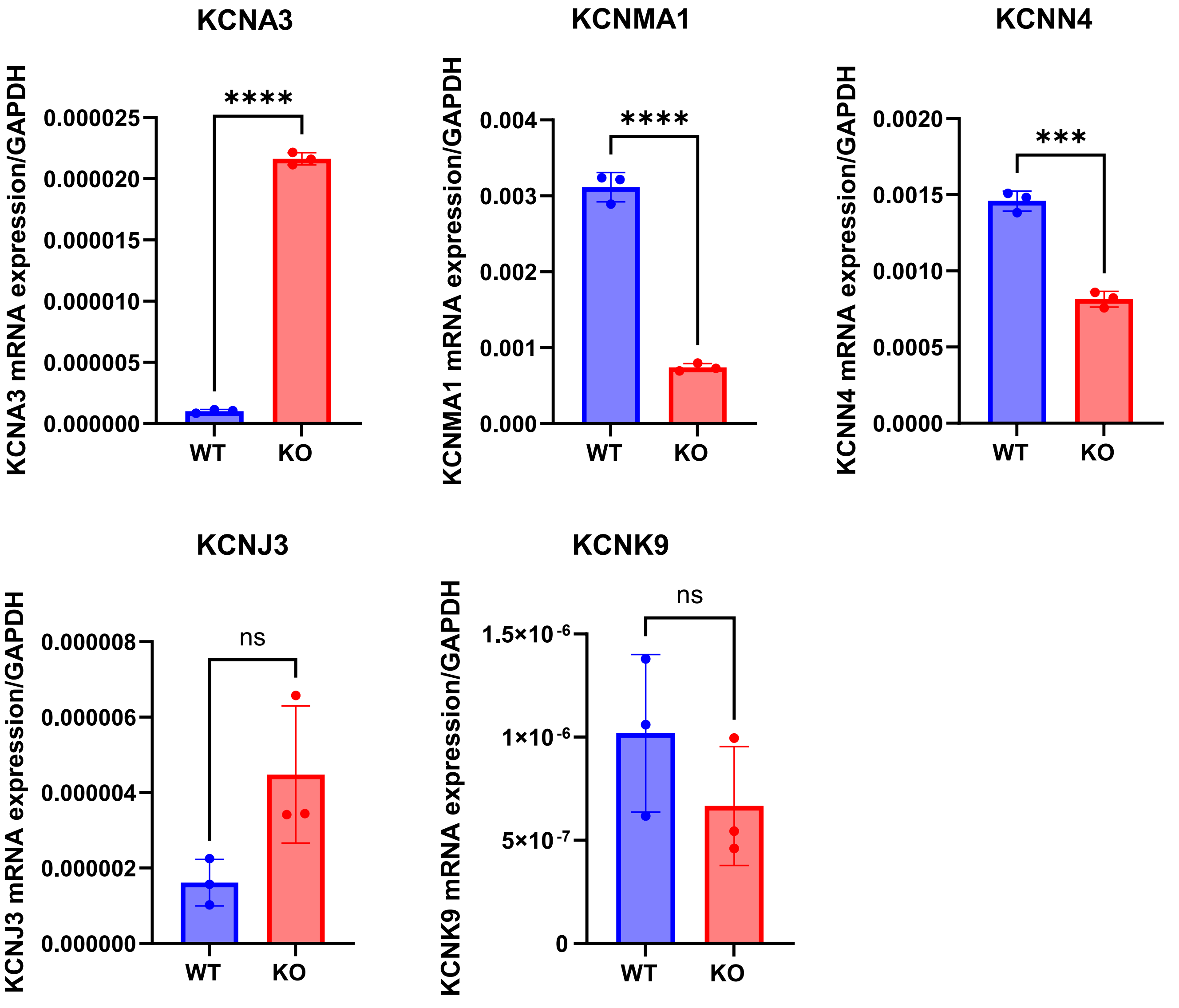

Supplementary Fig. 3. Expression profile of genes encoding potassium channels involved in oncogenesis in WT and EAG1-KO A549 cells. Bar graphs of KCNA3, KCNMA1, KCNN4, KCNJ3 and KCNK9 mRNA expression for WT and EAG1-KO A549 cells relative to GAPDH expression assessed with RT-qPCR. Data represent three repeated experiments for each cell line. The data are presented as mean ± SD. Unpaired Student's t-test was used to determine statistical significance throughout the figure (**** $P < 0.0001$; *** $P < 0.001$; ns $P > 0.05$).

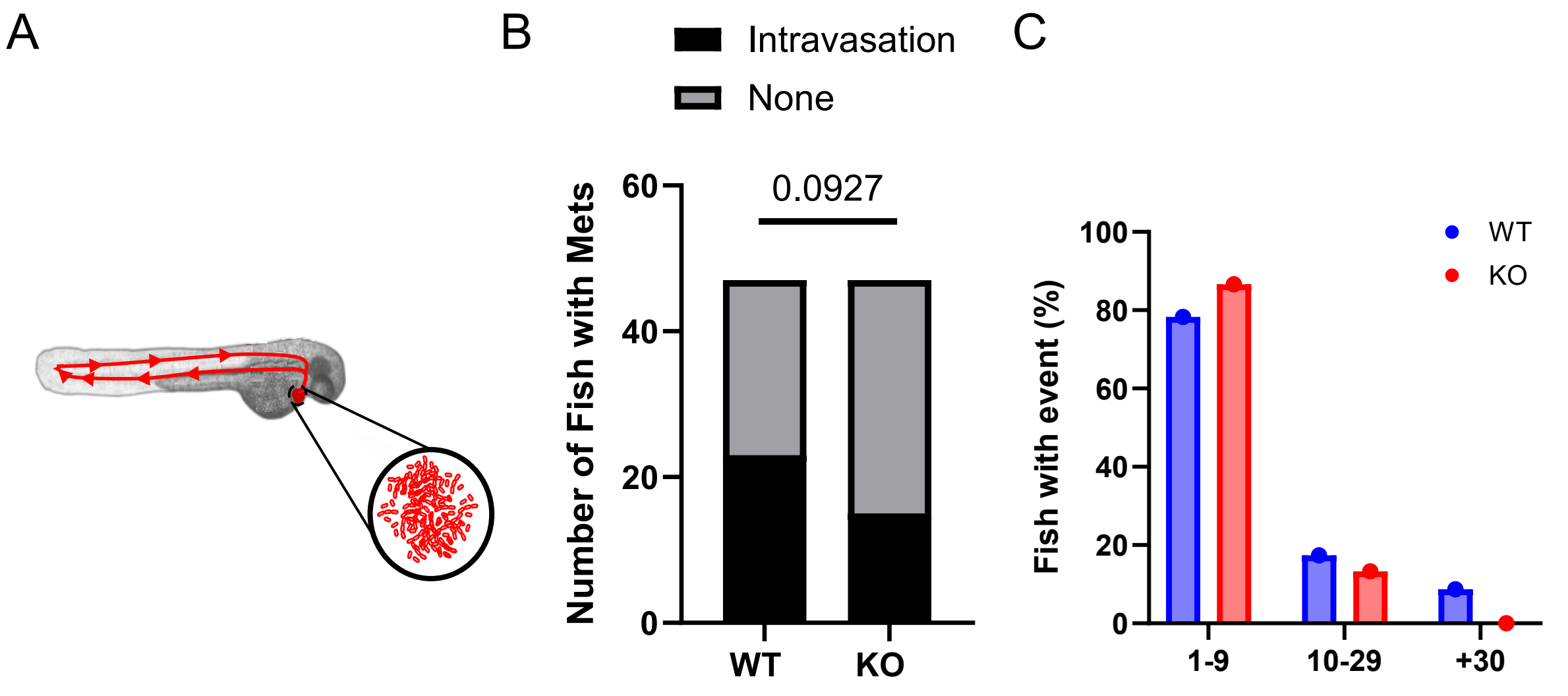

Supplementary Fig. 4. EAG1 knockout decreases aggressive intravasation. (A) Schematic of the experimental design, where DiI-labeled cells are injection into the Precardiac space of 2 dpf zebrafish with GFP-expressing endothelial cells. If cells manage to intravasate, they travel into circulation and end in the tail region. (B) Stacked bar graph showing the number of fish with at least 1 intravasating cell (black) and no intravasating cells (grey) for A549/WT xenografts and A549/EAG1-KO xenografts. Number of zebrafish embryos N = 47 for both A549/WT and A549/EAG1-KO xenografts. A CHI-test was used to determine significance. (C) A bar graphs showing the percentage of fish with 1–9, 10–29 metastases, or >30 metastasized cells, calculated from the total number of fish that developed any metastases, for WT (blue) and A549/EAG1-KO (red) xenografts.

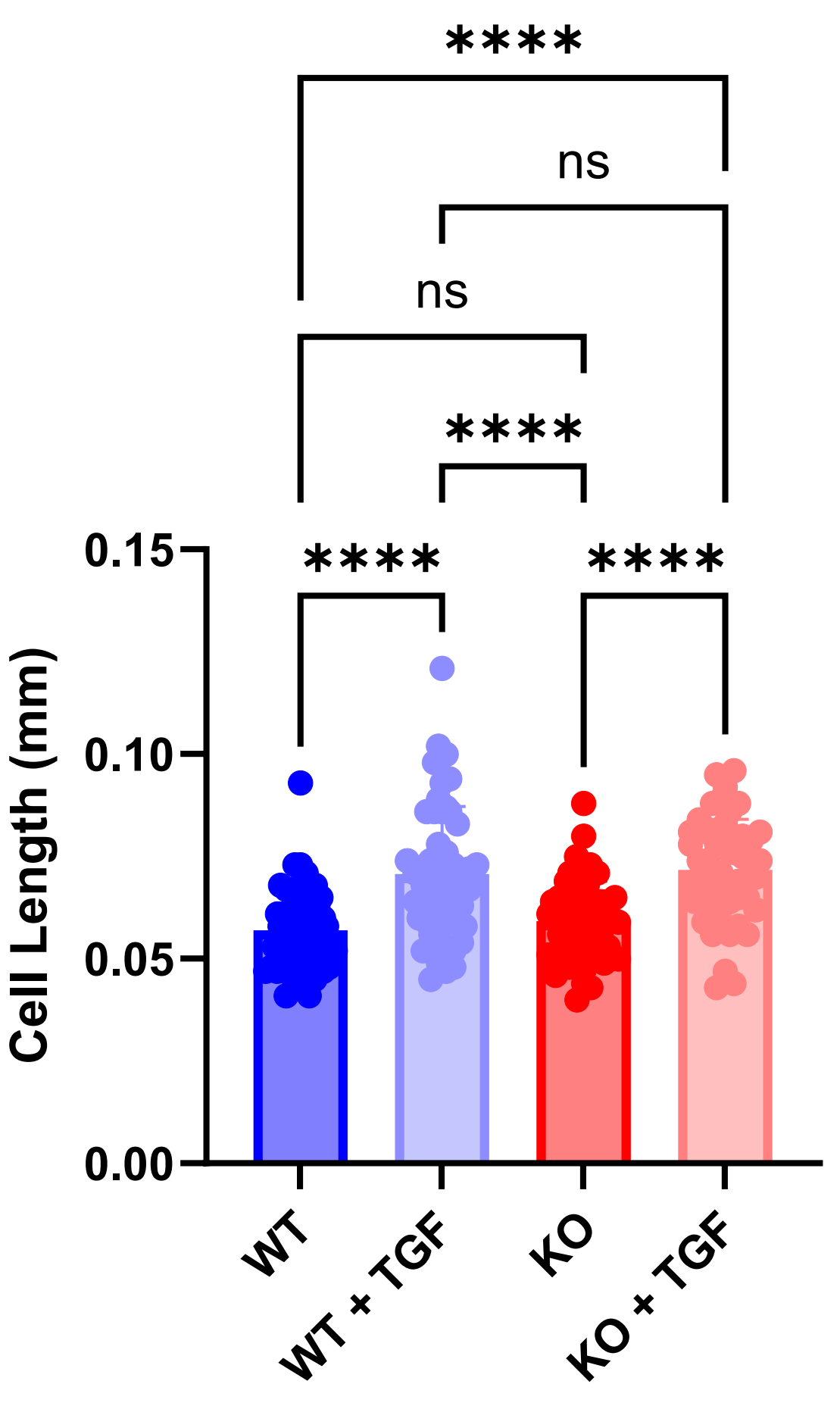

Supplementary Fig. 5. Quantitative analysis of cell length. The length of 50 different WT or EAG-KO A549 cells cultured with or without TGF-β, as indicated. The data are presented as mean ± SD. One-way ANOVA analysis was used to determine statistical significance (**** $P < 0.0001$; ns $P > 0.05$).

Allele Table

Sample Name	D5S818	D13S317	D7S820	D16S539	vWA	TH01	AMEL	TPOX	CSF1PO	D3S1358	D21S11	D18S51	Penta_E	Penta_D	D8S1179	FGA
	Reference STRs									Additional STRs						
WT A549	11<br>11	11<br>11	8<br>11	11<br>12	14<br>14	8<br>9.3	X Y	8<br>11	10<br>12	16<br>16	29<br>29	14<br>17	7<br>11	9<br>9	13<br>14	23<br>23
EAG1-KO A549	11<br>11	11<br>11	8<br>11	11<br>12	14<br>14	8<br>9.3	X Y	8<br>11	10<br>12	16<br>16	29<br>29	14<br>17	7<br>11	9<br>9	13<br>14	23<br>23
A549 (ExPASy)	11, 11	11, 11	8,11	11,12	14, 14	8,9.3	X, Y	8,11	10,12	16, 16	29, 29	14,17	7,11	9, 9	13,14	23, 23

Electropherograms for WT A549

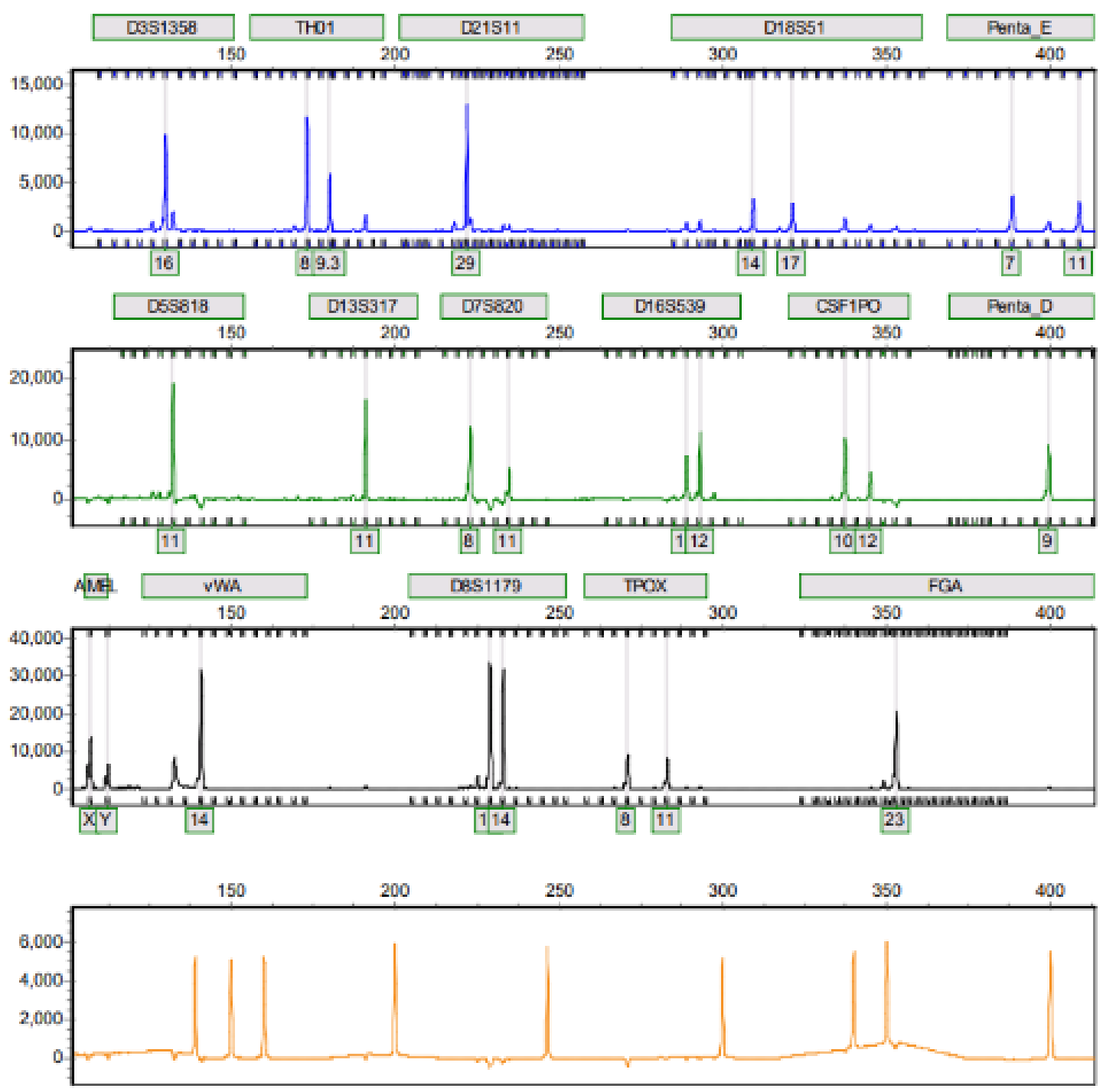

Electropherograms for EAG1-KO

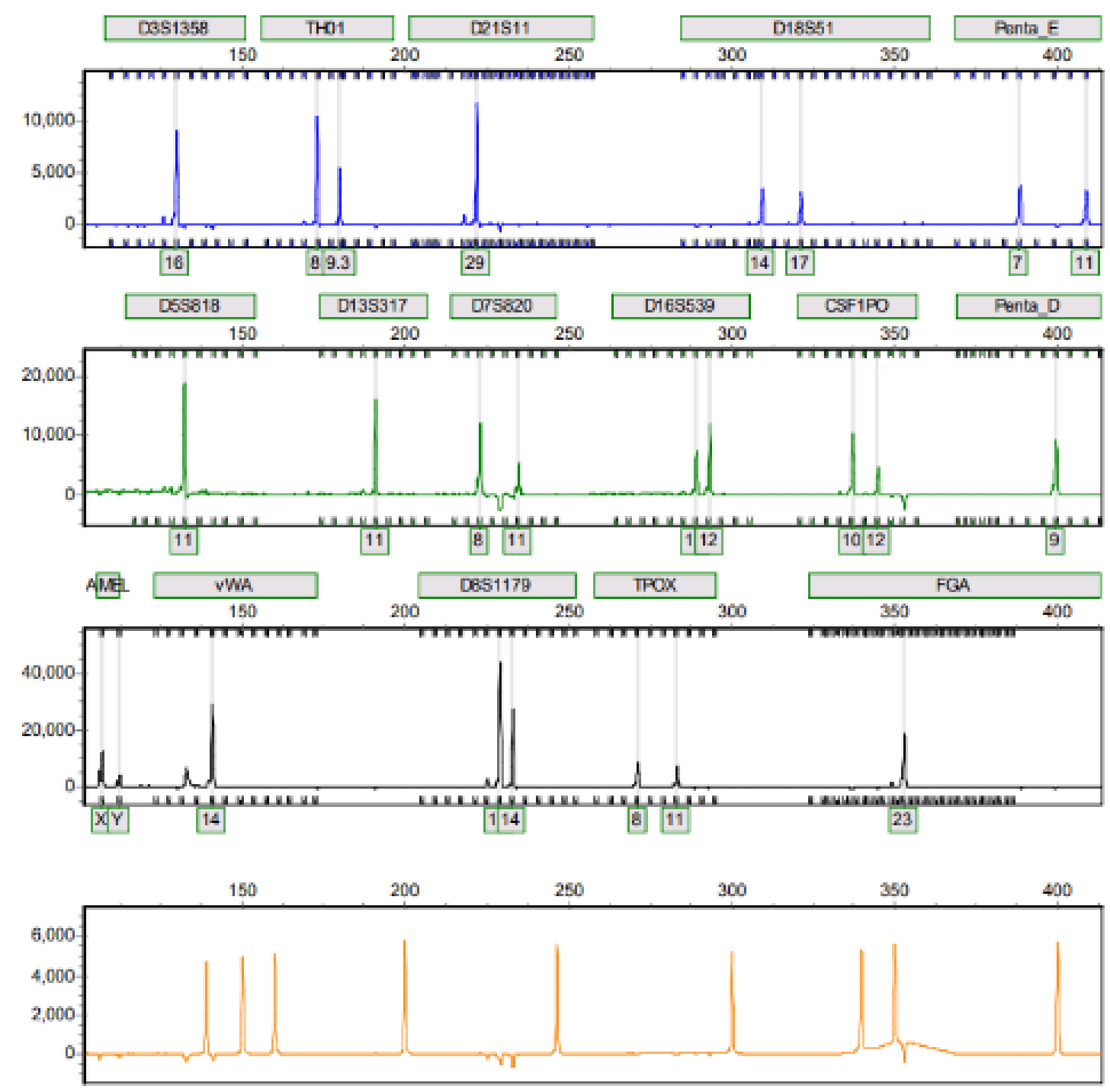

Supplementary Fig. 6. Confirmation of WT and EAG1-KO A549 cell identity using STR profiling. The allele table and electropherograms for WT and EAG1-KO cells, as indicated.
